# Uncovering complex microbiome activities via metatranscriptomics during 24 hours of oral biofilm assembly and maturation

**DOI:** 10.1186/s40168-018-0591-4

**Published:** 2018-12-06

**Authors:** Anna Edlund, Youngik Yang, Shibu Yooseph, Xuesong He, Wenyuan Shi, Jeffrey S. McLean

**Affiliations:** 1 Genomic Medicine Group, J. Craig Venter Institute, 4120 Capricorn Lane, La Jolla, CA 92137 USA; 2 National Marine Biodiversity Institute of Korea, 75, Jansang-ro 101beon-gil, Janghang-eup, Seocheon-gun, Chungcheongnam-do 33662 Korea; 3 Department of Computer Science, University of Central Florida, 4328 Scorpius Street, Orlando, FL 32816 USA; 4 The Forsyth Institute, Cambridge, MA 02142 USA; 5 Department of Periodontics, University of Washington, Seattle, WA 98195 USA

**Keywords:** /oral biofilm/ biofilm succession/ community function/ low pH/ metatranscriptomics/ metagenomics/ *Streptococcus*/ *Lactobacillus*/ *Veillonella*/ *Granulicatella*

## Abstract

**Background:**

Dental plaque is composed of hundreds of bacterial taxonomic units and represents one of the most diverse and stable microbial ecosystems associated with the human body. Taxonomic composition and functional capacity of mature plaque is gradually shaped during several stages of community assembly via processes such as co-aggregation, competition for space and resources, and by bacterially produced reactive agents. Knowledge on the dynamics of assembly within complex communities is very limited and derives mainly from studies composed of a limited number of bacterial species. To fill current knowledge gaps, we applied parallel metagenomic and metatranscriptomic analyses during assembly and maturation of an in vitro oral biofilm. This model system has previously demonstrated remarkable reproducibility in taxonomic composition across replicate samples during maturation.

**Results:**

Time course analysis of the biofilm maturation was performed by parallel sampling every 2–3 h for 24 h for both DNA and RNA. Metagenomic analyses revealed that community taxonomy changed most dramatically between three and six hours of growth when pH dropped from 6.5 to 5.5. By applying comparative metatranscriptome analysis we could identify major shifts in overall community activities between six and nine hours of growth when pH dropped below 5.5, as 29,015 genes were significantly up- or down- expressed. Several of the differentially expressed genes showed unique activities for individual bacterial genomes and were associated with pyruvate and lactate metabolism, two-component signaling pathways, production of antibacterial molecules, iron sequestration, pH neutralization, protein hydrolysis, and surface attachment. Our analysis also revealed several mechanisms responsible for the niche expansion of the cariogenic pathogen *Lactobacillus fermentum.*

**Conclusion:**

It is highly regarded that acidic conditions in dental plaque cause a net loss of enamel from teeth. Here, as pH drops below 5.5 pH to 4.7, we observe blooms of cariogenic lactobacilli, and a transition point of many bacterial gene expression activities within the community. To our knowledge, this represents the first study of the assembly and maturation of a complex oral bacterial biofilm community that addresses gene level functional responses over time.

**Electronic supplementary material:**

The online version of this article (10.1186/s40168-018-0591-4) contains supplementary material, which is available to authorized users.

## Background

Supragingival biofilms are remarkably complex structures, with well characterized stages of development, initiated by the attachment of bacteria to the tooth surface [1]. Bacterial community members in these structures are highly adapted to the oral environment and are not commonly found outside of the mouth. Understanding the processes underlying the assembly of complex oral biofilm communities is of high importance for maintaining a healthy microbiome composition and learning how dysbiosis occurs. Oral bacteria and their metabolites can interact directly with human host cells, both as protective barriers against pathogen invasion [2], and as causative agents of oral diseases [3, 4] (e.g. caries and periodontal disease), as well as systemic diseases (e.g. type-2 diabetes and cardiovascular disease) [5, 6]. The overall composition of the climax community of plaque is diverse, with many species being detected at individual sites [7]. Earlier studies, using deep sequencing of 16S rRNA gene fragments, have identified a total of > 700 bacterial taxa, of which some are currently unculturable [3, 8, 9]. The taxonomic succession of select early biofilm colonizers has been well documented, which has led to an increased understanding of their spatiotemporal development [10–12]. Once plaque forms, its species composition at a site remains relatively stable, in spite of regular minor environmental stresses, e.g., from dietary components, oral hygiene, host defenses, diurnal changes in saliva flow, etc. Interactions within oral biofilm communities can be cooperative or competitive, and bacteria have evolved highly defined pathways to sense and adapt to cues from neighboring species [13]. Up to 80% of the initial colonizers have been reported as streptococci*,* which are prolific producers of a sticky exopolysaccharide matrix, a highly stable multicellular biofilm structure, that not only promotes a rich supply of nutrients, but also enables accumulation of chemicals for communication exchange with other bacteria. Oral streptococci are also well known for their production and secretion of antimicrobial compounds (e.g. hydrogen peroxide, bacteriocins), which are of ecological significance. Major signatures of oral streptococci include the consumption of carbohydrates and the rapid secretion of L-lactate and hydrogen peroxide (H_2_O_2_), that can accumulate to high (millimolar) concentrations within mixed-species biofilms [14]. These capacities render streptococci extremely competitive by respectively limiting carbon source availability, and causing oxidative stress to surrounding microbes. Therefore, in order for secondary biofilm colonizers to survive in a close proximity to these pioneering colonizers they have to be resistant to streptococcal produced antimicrobial compounds, as well as being highly competitive with regards to carbon source utilization, or harbor the capacity to utilize end-products from streptococci metabolism, such as L-Lactate. A known example of such organisms are bacteria belonging to the *Veillonella* genus, which carry H_2_O_2_ resistance genes [15], and can utilize L-lactate as a sole carbon source [16].

Much of our current knowledge of the oral microbiome is derived from studies of in vitro models with 2–10 species [17–19], or is inferred from culture independent studies, looking only at taxonomic changes in populations [20–23]. In this study, our goal was to uncover oral bacterial behaviors at the transcriptional level, with high temporal resolution during community assembly, from early community establishment to later stages of maturation in response to change of the overall pH of the biofilm due to fermentation of carbohydrates. By applying our well characterized in vitro biofilm model system [24–26] that maintains 60–80% of the bacterial operational taxonomic units (OTUs) (approximately 130 OTUs in total) in the original saliva inoculum [25], we were able to monitor both the collective community gene activity as well as the activity of individual bacterial taxa. Importantly, these studies show that in vitro community assembly over time is highly regulated and reproducible across samples, resulting in near identical taxonomy and abundances at 16 h of growth [25, 26]. We also observed blooms in dental caries related taxa at later stages of growth when the pH dropped below 5.5 [25]. In an earlier study, using same model system, we conducted parallel metatranscriptomics (MT) and global metabolomics (extracellular and internal) using gas chromatography mass spectrometry (GC-MS) to gain a deeper understanding of the interplay between gene expression and core metabolites, and to understand the homeostatic processes of dental plaque during the rapid transition from neutral pH to acidic pH, and the health associated pH-recovery [26]. We noted that replicate samples were also highly correlated with respect to changes in gene expression [26]. In addition, using the same model and employing liquid chromatography mass spectrometry (LC-MS), we showed that secreted peptidic small molecules (PSMs), which many times represent important signaling molecules (e.g. cyclic peptides and bacteriocins), can vary over time as the biofilm community develops despite minor taxonomic changes [27]. Taken together, these results suggested that metabolic switches exist throughout community development, and that bacterial functions can change more frequently than taxonomic changes over a relatively short period of time (24 h).

In order to understand the transcriptional dynamics and shed light on the changes that occur across the different stages of oral biofilm community assembly, including key events like taxa blooms, we conducted comparative meta-OMICS analyses of both DNA (metagenomics) and mRNA (metatranscriptomics). Biofilm samples were collected over a 24-h time period at two to three hour intervals. As the biofilm developed, we identified taxa blooms and dramatic changes in expression of genes previously associated with antibacterial activities, protein hydrolysis, cell-to-cell communication, virulence as well as genes involved in pH-neutralization. Interestingly, the significant gene expression changes we observed did not co-occur with changes of community taxonomy but with biofilm development and acidification of the environment. Moreover, the abundance of several bacterial taxa were discordant when comparing DNA- and mRNA- deep sequence read frequencies throughout the study period, suggesting that the most abundant taxa were not necessarily the most transcriptionally active taxa.

## Methods

### Growth media for biofilm establishment

SHI medium was prepared following the protocol of Tian and colleagues [24]. Detailed SHI medium preparation protocols are available at https://research.dental.uw.edu/mclean/wp-content/uploads/sites/3/2015/02/Shi-Media-Preparation.pdf and in the Additional files section.

### Sample collection and incubation conditions

Saliva samples were collected and pooled from six healthy subjects, age 25–35 years as described previously [24, 25]. None had active caries or were being treated for any systemic disease or taking any prescription or non-prescription medications. Consents from study subjects, including consent to participate in the study and consent to publish findings from saliva samples were obtained. Subjects were asked to refrain from any food or drink 2 h before donating saliva and to spit directly into the saliva collection tube; 5 ml saliva was collected from each person. The saliva samples were immediately transferred to the laboratory and centrifuged at 6000 g for 5 min to separate the bacterial cells from debris and eukaryotic cells. Supernatants from the six subjects were pooled and transferred to sterile 2 ml cryovial tubes and mixed with glycerol (20%) for long term storage in − 80 °C. 10 μl of the pooled samples were inoculated into SHI medium [24] within a sterile 24-well and sucrose was added at 27.8 mM concentration. Samples were incubated for 24 h at 37 °C in anaerobic conditions. Cell-free saliva was also used for coating wells prior to growing the biofilms (for protocol see the Additional files section). Sample collection of biofilms was performed inside the anaerobic chamber at 10 different time points during the 24-h growth period for mRNA and DNA isolation as described below.

### pH monitoring of in vitro biofilm growth medium

After seeding the saliva samples in SHI medium, pH was monitored in replicate pH-designated incubation wells and measured by using a pH Laboratory Electrode (EW-05990-65, Cole-Parmer, Court Vernon Hills, IL). pH measurements were collected in parallel with sample collection. pH measurements were collected in the growth media adjacent to the biofilms from growth wells that were not used for nucleic acid extraction.

### mRNA and DNA isolation, library preparation and sequencing of in vitro biofilms

Detailed protocols for mRNA and DNA extraction, sequencing library preparation and quality filtering of mRNA and DNA reads are provided in the Additional files section. In brief, samples for mRNA and DNA isolation were collected after removing 0.5 mL of the spent SHI medium and adding 2 volumes of RNAProtect (QIAGEN Sciences Inc. USA, Valencia, CA) to each growth well. After adding RNAProtect, biofilms were transferred with sterile and RNAse/DNase-free pipet tips (Thermo Fischer Scientific, Carlsbad, CA) to sterile and RNase/DNase-free Eppendorf tubes (Thermo Fischer Scientific). Each sample was then divided into two separate tubes; one for DNA extraction and one for RNA extraction. Replicate libraries were prepared from separate growth wells, representing biological replicates. 2–3 biological replicate mRNA libraries were prepared per growth stage, while 2 biological replicates were prepared for the DNA libraries. Sequencing of mRNA and DNA was carried out at the (J. Craig Venter Institute) JCVI Joint Technology Center (JTC) by using an Illumina NextSeq 500 platform (San Diego, CA, USA) (150 bp paired end reads). mRNA and DNA sample concentrations were normalized at JTC (JCVI technical service core) prior to sequencing.

### Read mapping of DNA and mRNA using burrows wheeler aligner (BWA)

Our previously generated oral reference genome database [26], which represents the highly diverse oral microbiome and that was constructed with annotated and full-length genomes (reference set) representative of 384 bacterial taxa was applied as a reference for mapping of mRNA and DNA read libraries, respectively. The absence of human reads in DNA sequencing libraries was confirmed by using the KneadData pipeline, available at https://bitbucket.org/biobakery/kneaddata. DNA and mRNA reads were mapped by using the BWA-MEM algorithm and default settings [28]. GABE, an alternative implementation of the expectation-maximization based GRAMMy framework [29], was applied to compute the relative abundances of genomes from the DNA read mapping data. GABE was created to allow read mapping files (i.e. sam formatted files) as input for analysis in GRAMMy. GABE uses the mapped reads to generate the relative abundance estimates of the genomes in the reference set. These estimates are genome-length normalized. GABE was implemented as a multi-threaded program for computational efficiency. This implementation is available at https://github.com/syooseph/YoosephLab/tree/master/gabe. Bacterial reference genomes that provided the highest coverage of mRNA reads were selected for individual genome analysis, which enabled comparative studies of genome activities across pH stages. Mapping counts for metatranscriptomes were normalized with the DESeq model that takes into account both technical and biological variability and is not biased towards gene length, GC content and dinucleotide frequencies which other known RNA-Seq normalization models can be [30]. Detailed processing of the mRNA- and DNA-read mapping analyses is available in Additional file 1. Ratios between mRNA and DNA read abundances for each genome were estimated by comparing growth stages from the BWA mapping events above. Ratios above 2 were considered indicative of higher genome transcription activity but lower genome abundance. These ratios were based on average values, calculated from two replicate DNA sequence libraries, and three replicate mRNA sequence libraries, except mRNA libraries representing six hours, 21 h, and 24 h of growth, which were represented by two replicates each. To visualize major difference of mRNA and DNA ratios, reference genomes, which showed the highest differences were included in a bar graph and heat map comparison using GraphPad Prism v. 6.0 h for Mac OSX (GraphPad Software Inc., La Jolla, CA). To achieve additional perspectives of the relative abundance of bacterial taxa in the communities we employed the Metagenomic Intra-Species Diversity Analysis System (MIDAS) pipeline [31]. This pipeline employs read mapping to a database of phylogenetic marker genes representing 5952 bacterial species. Hierarchical cluster analysis was performed on the resulting relative abundance estimates using z-scores, Pearson correlation distance and the Heatmapper program [32]. Taxa were included in the hierarchical cluster analysis if they contributed with ≥0.5% to the total number of reads at any given time point.

### Community transcriptomic profiles

To gain a deeper understanding of gene expression responses associated with community assembly we applied the non-redundant ORF-reference dataset [26] to classify and quantify mRNA reads from all 21 mRNA-libraries at the gene level. The ORF dataset, which was generated in our previous study [26] consists of 2,288,459 unique KEGG-annotated ORFs representing genes extracted from several hundred sequenced oral bacterial genomes, de novo assembled genes from our previously described metagenomes from the in vitro grown biofilms as well as de novo assembled cDNA reads [25, 26]. The software used and their parameters are described in detail in a previous study [26]. A protocol and references are also presented in Additional file 1.

### Growth estimates of two key-community members by marker gene analysis

Changes in relative bacterial growth across pH stages were analyzed based on normalized expression of cell division genes. GABE-normalized DNA read copy numbers from a subset of 20 single copy marker genes [33] were extracted from the MG data. Correlations between relative marker gene activity and relative gene abundance were analyzed by linear regression using GraphPad Prism v. 6.0 h for Mac OSX for two key-community member representatives of *S. parasangunis* ATCC15912 and *L. fermentum* IFO 3956. Cell division genes (*ftsZ* and *ftsA*) were plotted on the x-axis against several ribosomal encoding genes, an acetyltransferase encoding gene and a GTP binding *lepA* encoding gene (y-axis). *P*-values and *R*^*2*^ values were calculated for each correlation.

### Biosynthetic gene cluster mining and mRNA read mapping

Biosynthetic gene clusters (BGCs) encoding small molecules were searched in 31 of the most active genomes in the community by using the antiSMASH v. 3.0 pipeline [34]. This resulted in the identification of 130 BGCs. Next, all MT sequence reads were aligned against the 130 BGCs using the BWA-MEM algorithm with default parameters. We used custom Perl script to process the sam files to extract the number of reads mapped to each BGC in each sample separately. Sequence read counts for each BGC was then summed up. The count file was normalized using DESeq pipeline as described previously. BGC were considered as active (expressed) if at least half of the core genes within a BGC were detected by a minimum abundance of 10 reads.

## Results and discussion

**Fig. 1:**
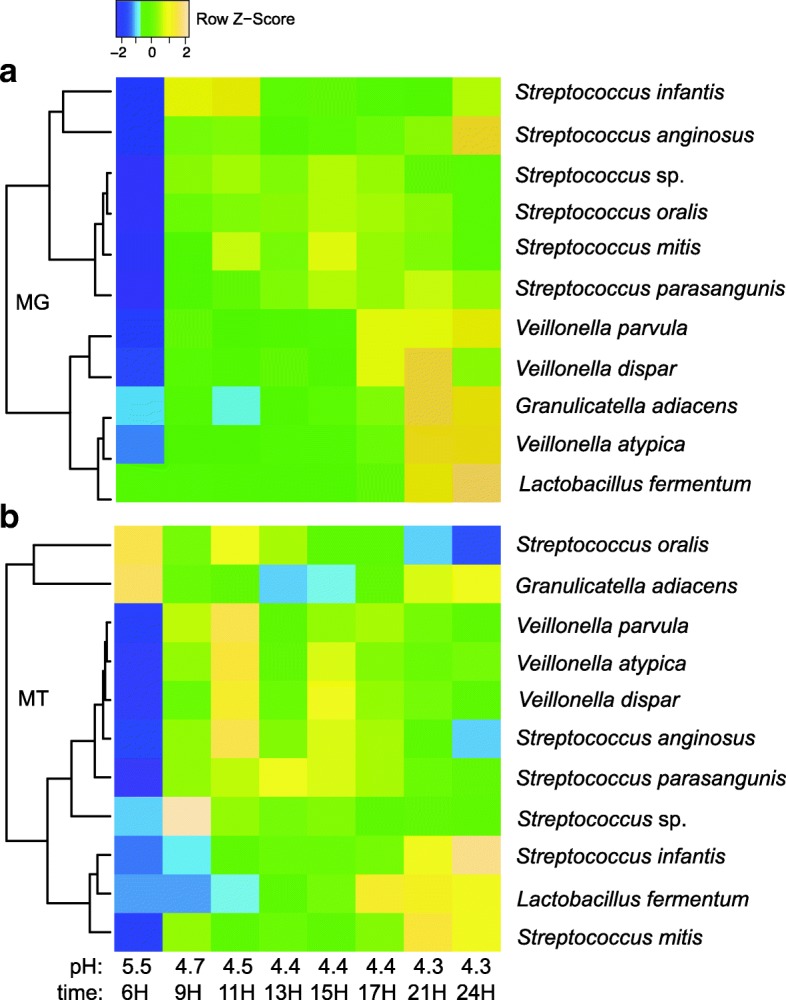
Hierarchical cluster analysis of key bacterial taxa relative abundances in the biofilm community obtained by analyzing DNA (**a**) and mRNA (**b**) reads and averaging the biological replicates with the Metagenomic Intra-Species Diversity Analysis System (MIDAS) pipeline. Pearson correlation was used as distance measure. Color gradients correspond to z-score values calculated from normalized abundance values. Taxa were included in bar graph if they contributed with ≥0.5% to the total number of sequence reads at any given time point between 6 h and 24 h of biofilm growth. Detailed bar graphs based on BWA-MEM read mapping results and DESeq and GABE normalization, which also include the biological replicates and less abundant taxa are visualized in Additional file 2: Figure S1

As of today, most of our understanding of oral biofilm succession has been obtained by applying taxonomy based temporal tracking of relative abundances using the bacterial 16S rRNA gene. One of our goals within this study was to move beyond limited descriptive taxonomy measures to deepen our understanding on the expressed bacterial functions during oral biofilm community assembly. Knowledge of molecular mechanism that shape bacterial community function will contribute to an increased understanding of key ecological processes that are important both in maintaining health as well as in disease initiation and progression. By continuing to study our previously well characterized in vitro biofilm model system [26] using metatranscriptomics and metagenomics analysis approaches, we were able to obtain a more mechanistic understanding of temporal metabolic processes, both at the community and species level during biofilm assembly. Each model system has strengths, limitations and difficulties and a major challenge, when using any oral model system, is to maintain a representative diversity of the indigenous oral microbiome. This was specifically addressed in previous studies where we developed a growth medium (SHI medium) that supported a remarkably high number of oral taxa in vitro from a small number of human saliva samples, which were inoculated and grown as biofilms [24, 25]. A limitation of using a pool of saliva samples deriving from several individuals as inoculum in the in vitro model system is that the native community integrity is lost as the samples are mixed together. Each study subject most likely harbored a unique saliva microbiome, which may not be accurately reflected from a community diversity perspective here. Clearly, in future studies it is important to address how a single person’s microbiome evolves taxonomically and metabolically over time in *our* in vitro model and to what extent species within a native community accomplish certain functions only in their native setting as compared to when they are mixed with other community members from other individuals. Regardless, as seen with in vivo communities, the resulting in vitro biofilm communities show a remarkable reproducibility in taxonomic composition across replicate samples during maturation in a mechanism that is not yet understood. Here, we addressed functional changes by applying a two- to three-hour time interval sampling regimen to capture gene expression during initial colonization and throughout the transition to a more mature plaque, which rapidly acidified the environment through sugar metabolism (caries disease-like conditions). Bacterial biofilm communities were seeded from a saliva pool collected from six healthy adults in the same growth medium as mentioned above [24, 25], in saliva coated growth-wells, and incubated for 24 h. In line with our previous oral in vitro biofilm studies [25–27], saliva was chosen as inoculum for this model since the initial binding and subsequent biofilm assembly on a tooth surface is initiated by early colonizing bacteria found in saliva, which bind to the newly deposited acquired pellicle containing host components [35]. Biofilms in the in vitro system mature over time and in parallel, bacterial biomass and fermentation products increase [24–26]. Metagenomic (MG) libraries were generated and analyzed from all time points, starting at zero hours of incubation, to 24 h. Metatranscriptomic (MT) mRNA libraries were generated from samples starting at six hours of biofilm growth to 24 h. Due to the absence of biofilm formation at time point zero (starting point of incubation) and the minimal formation of biofilms after three hours of growth, high quality mRNA libraries could not be obtained from these two first stages. Therefore, we focused on addressing the gene expression activities in the in vitro biofilms between six (pH 5.5) and 24 h (pH 4.3) of growth. Taxonomic stability at the DNA level was observed between six and nine hours of biofilm establishment for a broad number of taxa, when pH had dropped to 5.5 and 4.7, respectively (Additional file 2: Figure S1). A few examples of temporal responses in relative abundance of DNA and mRNA sequence reads, representing key genomes are visualized in detail Fig. 1. Notably, the cariogenic and lactic acid producing *L. fermentum* increased in relative abundance both at the mRNA and DNA levels at later time points, which suggests its role as a secondary colonizer. Reproducibility of mRNA and DNA libraries from biological replicate samples (i.e. biofilms growing in different growth wells) was high (Additional file 2: Figure S1), and mRNA sequence reads from all growth stages resulted in deep genomic coverage of the most abundant taxa in the community (e.g. Additional file 3: Tables S1, Additional file 4: Table S2, Additional file 5: Table S3, Additional file 6: Table S4, Additional file 7: Table S5 and Additional file 8: Table S6). A significant amount of the mRNA sequence reads (87–95%) could be mapped back to our reference genome database (Additional file 9: Table S7), showing that only a small fraction of the metatranscriptome remained unassigned.

### Overall transcription abundance estimates of genomes

**Fig. 2:**
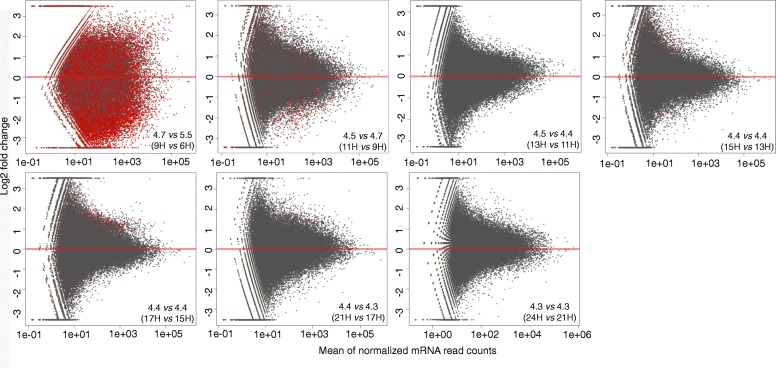
MA plots from DESeq analysis showing differences in gene transcription activity between pH stages. Log_2_ fold changes are presented on y-axes, while means of the normalized read counts are presented on x-axes. Red horizontal lines indicate zero-fold change, and red dots in individual graphs represent all genes independent of fold change cutoffs that changed significantly (fdr corrected *p*-value < 0.05)

A large fraction the mRNA reads in the study were mapped to *Streptococcus* genomes (Additional file 2: Figure S1, Additional file 10: Table S8) and at six and nine hours of growth, when pH dropped from 5.5 to 4.7 several community members responded with an overall increase in gene transcription activity at the genome level, e.g. *S. parasanguinis* ATCC 15912 (2.4-fold), *S. parasanguinis* F0405 (3.0-fold), *S. vestibularis* F0396 (7.6-fold), *S. salivarius* JIM8780 (4.4-fold), *Veillonella* genomes (3–23.6-fold) and *Lactobacillus fermentum* IFO 3956 genome (3.9-fold) (See Additional file 11: Table S9). However, other genomes showed decreased activity, e.g. *Granulicatella adiacens* (3.8-fold)*, G. elegans* (2.4-fold), *L. salivarius* (1.9-fold), *S. pneumoniae* (1.9-fold) (See Additional file 11: Table S9). The total number of genes that were expressed in the community at the different growth stages ranged between 37,666 and 40,287 (Table 1). A major change in gene transcription for overall community members occurred when pH dropped from 5.5 to 4.7 as 29,015 genes were significantly impacted (fdr corrected *p*-value < 0.05), and either up- or down expressed (14,416 up, 14,599 down) (Fig. 2, Table 1). Differences in gene transcription activity decreased drastically between nine hours and 11 h of growth, i.e. only 1979 genes changed significantly (fdr corrected *p*-value < 0.05) when pH dropped from 4.7 to 4.5. Between 11 and 13 h, when pH changed from 4.5 to 4.4, 565 genes changed activity (Fig. 2, Table 1). Between 17 and 24 h, by comparing subsequent time points, only 27 genes changed, significantly in expression, which happened in the transition period where pH dropped from 4.4 to 4.3. Taken together, the results show that bacterial gene transcription activities within the in vitro biofilm community remained high throughout the 24 h of incubation and that activity changed dramatically at the gene level after exceeding pH 5.5 and decreasing to 4.7. Gene functions that changed most significantly during the 24-h growth period, and their taxonomic origin will be discussed in the following sections. It is important to note that the majority of the gene annotations we obtained here derive from homology searches using publicly available reference protein databases. Thus, the functions of most of these reference proteins have not been experimentally characterized therefore, many of the annotations we present in the discussion below represent putative functions. In addition, our reference collection includes many hypothetical proteins found in all microbial genomes to date.

**Table 1:** Gene expression comparisons between time points and pH stages based on DESeq-fold change calculations

Time point | pH stages hours (H)	Tot. No. genes transcribed	No. genes changed significantly ^a^	Up / down expressed genes ^b^
9H-6H | 5.5/4.7	40,287	29,015	14,416/14,599
11H-9H | 4.7/4.5	37,666	1979	976/1002
13H-11H | 4.5/4.4	37,942	565	361/204
15H-13H | 4.4/4.4	37,941	562	358/204
17H-15H | 4.4/4.4	38,295	398	238/160
17H-21H | 4.4/4.3	38,093	27	17/10
21H-24H | 4.4/4.4	38,522	0	0

^a^Number (No.) of genes that changed significantly (fdr corrected *p*-value < 0.05)

^b^Number (No.) of significantly up or down expressed genes (fdr corrected *p*-value < 0.05)

Initially, we analyzed overall gene transcription responses at the genome level throughout the 24-h growth-period and captured distinct patterns of activity within different bacteria. Briefly, we conducted read mapping with mRNA sequencing libraries from the different time points to our reference genome database (as described in the Methods section). A DESeq normalization approach was applied and relative abundance values (in percentage) of the total number of mRNA reads that mapped to each genome, at each time point were calculated. Based on these results it became clear that 22 genomes showed an overall increased expression pattern, up to 23.6-fold, at nine hours of growth when pH dropped below 5.5 (e.g. *S. parasangunis* and various *Veillonella* genomes) (Fig. 1, Additional files 10 and 11: Tables S8 and S9). After this pH drop, gene transcription activity remained relatively stable across time points for these particular genomes, revealing their unique adaptation to an extreme low-pH environment. A second major pattern was represented by a genome belonging to *L. fermentum*, which showed a dramatic increase in activity (50- to 200-fold upexpression of overall genes) below pH 5.5 (Fig. 1, Additional file 3: Table S1). A third pattern was represented by groups of bacteria whose transcriptional activity decreased as pH dropped below 5.5, but recovered slowly and increased gradually in transcription over time (e.g. *Granulicatella adiacens*) (Fig. 1, Additional file 11: Table S9). *G. adiacens* contributed with approximately 10% of total community mRNA reads at six hours of growth at pH 5.5, but this number dropped to approximately 1% as pH further dropped to 4.4 (See Additional file 10: Table S8). Then, at the later time points (between 21 h and 24 h), *G. adiacens* regained some of its transcriptional activity and contributed with approximately 3% to the total mRNA pool at the end of the incubation. By calculating fold changes of gene transcription associated with each genome, and performing hierarchical cluster analysis of relative abundance data we were able to identify at least three behaviors of gene transcription activities during biofilm establishment and maturation (Fig. 1, Additional file 11: Table S9). These three behaviors are likely reflecting different timing in colonization, niche expansion and adaptation to acidic pH. Being able to capture this information in a highly complex biofilm environment allows us to focus on ecologically important taxa, as well as genes and metabolic pathways that display significant temporal activities during biofilm maturation.

### Growth estimates of key-community members

Previous studies demonstrate how data on the sequence and abundance of conserved single copy genes in metagenomic data can be used to estimate organismal abundance in microbial communities [33, 36]. Here we applied a similar concept by analyzing the relative abundance of DNA-marker genes with the goal to understand if growth and cell division indices could provide a deeper understanding of behaviors of two community members with different colonization preferences, i.e. *S. parasanguinis -* an early colonizing commensal bacterium, and *Lactobacillus fermentum*, a cariogenic pathogen considered a late biofilm colonizer. An additional goal was to address if a metagenome view provides a realistic picture regarding key contributors in the community. By relying on DNA sequencing technology alone it is possible that low-abundance species with high gene/genome activity are overlooked, which potentially could lead to misinterpretation of both ecological and clinical data, e.g. highly active key-pathogenic species may be mistaken as less important due to their low DNA abundance.

To gain more specific knowledge of relationships between cell division and growth for the two key-community members *S. parasangunis* and *L. fermentum*, which showed highly different relative abundance profiles, we extracted the normalized DNA read mapping values for a subset of 20 single copy marker genes (e.g. ribosomal proteins) [33, 36] and cell division genes (e.g. *ftsZ* and *ftsA*) from each genome and time point. To address if cell division and growth correlated for the individual genomes we conducted linear correlation analysis between the two different groups of marker genes (See Additional file 12: Table S10). This analysis showed significant correlations (*p* < 0.001, See Additional file 12: Table S10) for both the *L. fermentum* and *S. parasangunis* genomes. However, the regression slope was more positive for *L. fermentum* (0.3–1.2) than for *S. parasangunis* (0.1–0.5) indicating that *L. fermentum* was growing faster than *S. parasangunis* from six hours of incubation when the MG measurements started (See Additional file 13: Table S10). This analysis suggests that *S. parasangunis* establishes growth in the community prior to six hours, which would be the case for an early biofilm colonizer. The data also supports that *L. fermentum* is a secondary colonizer that initiates growth later, as the environment becomes more favorable for its aciduric lifestyle. Elevated growth of *L. fermentum* could also be observed from the gene expression data as several genes involved in cell division (i.e. *ftsA*, *ftsH*, *ftsQ*, *ftsZ*) increased in expression below pH 5.5 (DESeq values ranged between 39 and 255) (See Additional file 3: Table S1). Prior to this growth stage no mRNA reads could be mapped to the *L. fermentum* genome.

**Fig. 3:**
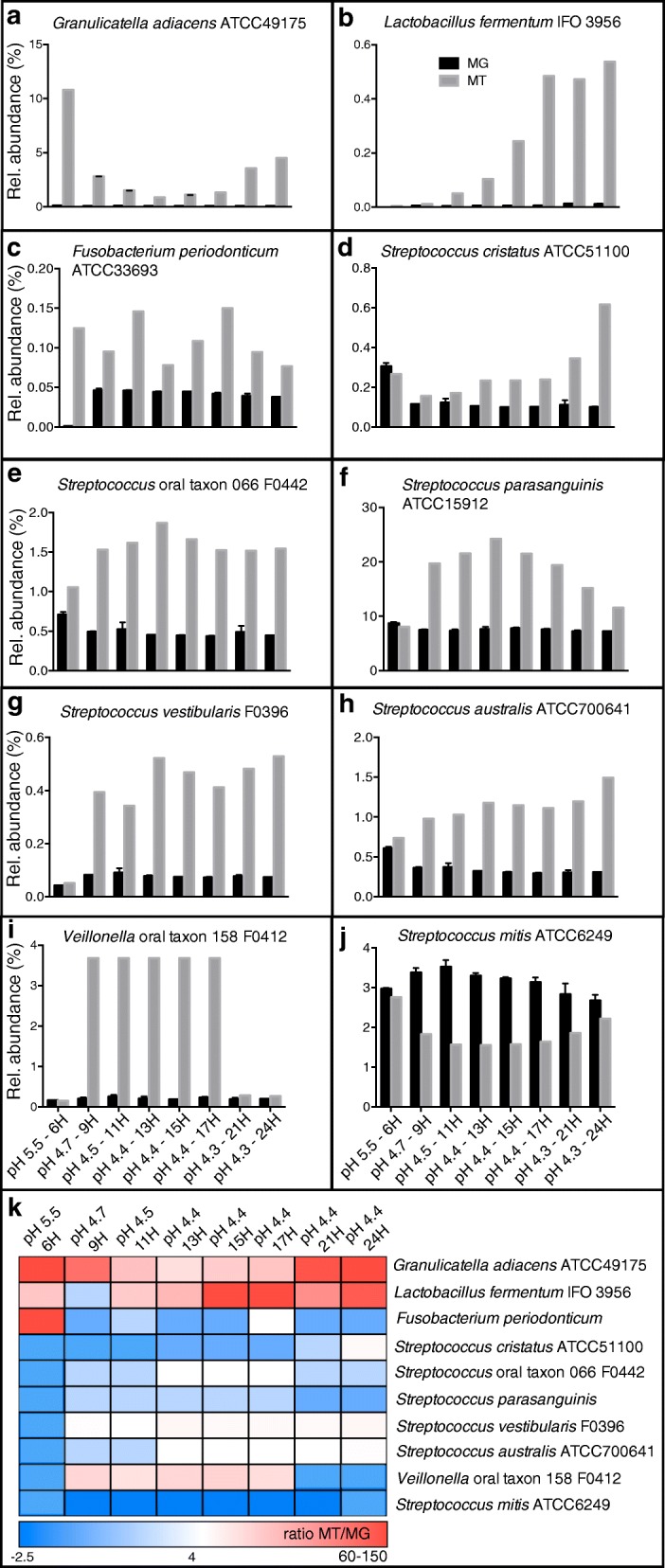
Relative abundance estimates from read mapping of bacterial DNA (black bars) and mRNA (gray bars). Ten bacterial genomes (**a**-**j**) with the largest differences between mRNA/DNA ratios were included. Panel k show a heat map of mRNA/DNA ratios for each genome across time points and pH stages. Ratios range between 2.5- and 150-fold differences

Our comparative analysis of mRNA and DNA genome (MT/MG) ratios, revealed that for some genomes, e.g. *G. adiacens*, *L. fermentum*, *Fusobacterium periodonticum*, *S. cristatus, Streptococcus* oral taxon 066, *S. parasangunis*, *S. vestibularis*, *S. australis*, *Veillonella* oral taxon 066, *and S. mitis*, the abundance of mRNA and DNA reads were disproportional, i.e. either relatively more mRNA reads were recruited to each genome than DNA reads, or vice versa (Fig. 3, See Additional file 13: Table S11). Also, the relative MT/MG ratio varied over time for the individual genomes (Fig. 3), suggesting that bacterial cells of the various populations were either in exponential or stationary growth phase, spending more or less energy on cell division (DNA synthesis and replication) and metabolic activity (mRNA synthesis and translation of mRNA). The highest mRNA/DNA ratios were observed for the *G. adiacens* and the *F. periodonticum* genomes, at six hours of growth with ratios of 108 and 159 respectively, indicating that these bacteria became highly metabolically active as pH dropped to 5.5 (Fig. 3-K, See Additional file 13: Table S11). *L. fermentum* responded in a similar fashion at 17 h of growth when pH had dropped to 4.4 (Fig. 3-K, See Additional file 13: Table S11). *Veillonella* oral taxon 158 also followed the same trend as its mRNA/DNA ratio increased between 16- and 19-fold as pH dropped below 5.5 (Fig. 3-K, See Additional file 13: Table S11). These results suggest that taxa, which are highly metabolically active and that play important ecological roles may be significantly underestimated when relying solely on DNA based technologies (e.g. MG and 16S rRNA gene analysis). The reason for the observed discrepancy could also be due to that some of the sequenced DNA represented dead and/or dormant bacteria, or that sequence read depth coverage was different between DNA and mRNA libraries (See Additional file 9: Table S7). We explored the latter and concluded that coverage of both DNA and mRNA reads across the individual genomes we included in the analysis is high (> 10X coverage) (See Additional file 10: Table S8). Therefore, we suggest it is of importance to be aware of that the choice of study-nucleic acid (DNA or RNA) can have an impact on how we identify ecologically and clinically important bacteria in complex communities. Translating knowledge of gene transcription activity to both the microbial ecological and clinical research fields, where DNA-based microbiome associations are the current norm, is an important undertaking and will result in a deeper understanding of which community members are actively interacting with one another, and with the human host.

### Changes in major biofilm community functions during community maturation

#### Lactate and hydrogen peroxide gene activities

In our previous multi-omic (metatranscriptomics and metabolomics) study of a similar in vitro biofilm model system, where we addressed sugar metabolism at the mature stage, we observed several characteristic genotypic and phenotypic traits associated with carbohydrate fermentation. In particular we noted significant increases in lactate production and excretion in parallel with high gene transcription activity of L-Lactate dehydrogenase genes harbored by several *Streptococcus* species [26]. Our metabolomics analysis also revealed the production of several other key organic acids as pH dropped [26]. Similar observations were made in this study during the community assembly as transcription activity of genes encoding D- and L-lactate dehydrogenases increased in parallel with a decreasing pH. Malate/L-Lactate dehydrogenases were highly expressed throughout the incubation period while pH decreased from 5.5 to 4.3 (between six hours and 24 h of incubation) (See Additional file 14: Table S12). By comparing fold changes in L-lactate dehydrogenase gene expression between six hours and 24 h (pH 5.5 and pH 4.3) of growth, we observed as much as a 721-fold upexpression of the gene in the *L. fermentum* genome (See Additional file 14: Table S12). Several *Veillonella* species showed upexpression of malate/L-lactate dehydrogenase encoding genes (up to 28-fold) at six hours of growth as pH dropped below 5.5, indicating that *Veillonella* are likely upregulating the enzyme to perform the reverse reaction to ferment lactate to pyruvate (See Additional file 15: Table S12). Lactate can also be oxidized by the L-lactate oxidase enzyme to form pyruvate. This reaction results in the removal of lactate and the subsequent production of pyruvate and toxic H_2_O_2_. In the in vitro biofilm community, lactate oxidation was carried out by nine different *Streptococcus* species (See Additional file 14: Table S12), and an increase in transcription of the *lctO* gene was observed at nine hours of growth at pH 4.7 (See Additional file 14: Table S12). The highest DESeq value for the *lctO* gene was 2289 normalized read counts, and was observed for *S. mitis* ATCC 6249, at 13 h of growth, indicating that the genes were expressed but not to the highest extent (See Additional file 14: Table S12). The *lctO* gene has previously only been observed expressed in aerobic environments; however, here it was unexpectedly identified during anaerobic incubation conditions. The product of lactate oxidation is pyruvate, which can then be further metabolized by bacteria that harbor the pyruvate oxidase enzyme, encoded by the *spxB* gene. This reaction results in the formation of acetyl phosphate and increased production of H_2_O_2_. Here, *spxB* genes were upexpressed in the same nine *Streptococcus* genomes as mentioned above (See Additional file 14: Table S12), suggesting that both lactate and pyruvate serve as substrates for the formation of the antimicrobial substance H_2_O_2_ in anaerobic growth conditions. In addition, the recently discovered Rnf-complex, which is also responsible for anaerobic lactate oxidation and the formation of pyruvate [37] was expressed at all time points by *Fusobacterium periodonticum* ATCC 33693, and *Stomatobacculum longum* (See Additional file 14: Table S12)*,* which are known secondary colonizing bacteria in mature plaque. Together these results imply a co-metabolic interaction where early colonizing community members produce lactate and also pyruvate, which then can be metabolized by later colonizing bacterial community members. Physical interactions between *Fusobacterium* and *Streptococcus* were previously identified to occur via the co-aggregating protein CmpA [38], and the adhesin protein RadD [39]. Here we show it is also highly likely that a close co-metabolic interaction exists between these two groups of bacteria. Both inter and intraspecies antagonism is evident in the expression data, which is specifically reflected by genes encoding H_2_O_2_ production (See Additional file 14: Table S12). H_2_O_2_ production in oral plaque is considered a major driver of oral biofilm community succession, and is critical for the prevention of pathogen invasion [15]. Oral streptococci are the main producers of H_2_O_2_ and also primary biofilm colonizers on teeth. In addition to the already mentioned *spxB* and *lctO* genes, bacterially produced H_2_O_2_ is catalyzed by the superoxide dismutase (SOD) enzymes (i.e. metalloenzymes that catalyze the dismutation of O_2_^−^ into H_2_O_2_ and O_2_). SOD genes that were highly upexpressed in the biofilm community belonged to *Streptococcus* and *Veillonella* community members (e.g. *Si, Streptococcus* sp. GMD4S, *Streptococcus* sp. M143, *S. mitis* bv. 2 str SK95, *S. mitis ATCC 6249, S. oralis* ATCC 35037, *S. oralis* Uo5, *S. tigurinus* 1366, Sp, *S. australis* ATCC 700641, *S. peroris* ATCC 70080, *V. parvula* DSM2008, *Veillonella* sp. 6_1_27, *Veillonella* sp. 3_1_44) (See Additional file 14: Table S12). Their expression remained high and even increased in some genomes as pH decreased further until incubation came to an end at 24 h. In parallel, genes that can detoxify H_2_O_2_ were expressed in streptococci and *Veillonella* (e.g. genes encoding Heam peroxidases and peroxidoxins were upexpressed five-fold in *Veillonella* sp. 3_1_44 and *Veillonella* sp. 6_1_27 genomes) (See Additional file 14: Table S12). Expression of H_2_O_2_-producing genes and H_2_O_2_-detoxifying genes remained high but varied in frequency in both *Streptococcus* and *Veillonella* groups throughout the incubation period. Our results here support previous findings of cooperative activities between these bacteria [40–43], as well as their joint fitness within the community through toxic H_2_O_2_-production.

#### Protease and chaperone gene activities

Other overall responses in key-genomes of the in vitro biofilm community was the upexpression of genes encoding proteases and chaperones (e.g. Clp proteases and the High-temperature requirement A protein, HtrA) (See Additional file 14: Table S12). *Clp* are *htrA* genes are ubiquitous among bacteria and are responsible for adaptation to multiple stresses by degrading accumulated and misfolded proteins [44]. For example, in cariogenic *S. mutans,* the HtrA and Clp proteins were previously shown to be responsible for acid tolerance and biofilm formation, which are major virulence attributes [44–49]. In this study, in the *S. parasangunis* and *S. infantis* genomes, the *htrA* gene increased in expression as pH dropped below 5.5 (See Additional file 14: Table S12). In addition, several genes encoding Clp proteases (e.g. *clpC, clpE, clpP, clpX*) were upexpressed (between two and 138-fold) in 14 different streptococci genomes and seven *Veillonella* genomes at low pH (See Additional file 15: Table S12). Based on our findings here, and previous studies [50], we suggest that both HtrA and Clp proteases in oral biofilms have highly important ecological roles in protection of low pH active bacteria. We propose that the expression of these genes were in particular important for the proliferation and survival of *S. parasangunis* and *S. infantis,* which showed increased activity at lower pH and later growth stages.

#### Expression dynamics across known and novel pH neutralizing pathways

To survive in a highly acidic environment, oral bacterial community members employ various enzymes for the neutralization of the low pH conditions. Here, we identified twelve putative pathways (i.e. several experimentally uncharacterized) that were highly upexpressed as pH dropped below 5.5 (See Additional file 16: Table S13). The activity of the well-known arginine deiminase (ADS) [51] pathway where arginine is oxidized to ammonia in three steps was specifically high in the *S. parasangunis* genome after 17 h of growth suggesting this may be a critical mechanism allowing *S. parasangunis* to thrive throughout the growth period (See Additional file 15: Table S13). Also, several bacteria employed only the last two steps in the ADS pathway (e.g. *L. fermentum*, *Solobacterium moorei* F0204, *Actinomyces* sp. oral taxon 181 and different *Veillonella* species), suggesting that pH neutralization also occurred via other pathways for these bacteria, or that they relied on other community members for the metabolic precursors required in the initial steps in the pathway. Streptococci and *Veillonella* shared some of the 12 expressed genes that encode ammonia-producing enzymes but they also expressed unique enzymes (e.g. serine deaminase in *S. parasangunis*, agmatine deiminase in streptococci genomes, adenine deaminase in *Veillonella* genomes). Interestingly, the nucleoside cytidine deaminase, which catalyzes the formation of ammonia and uridine was highly employed by 37 genomes representing five different genera (*Veillonella, Streptococcus*, *Granulicatella, Actinomyces,* and *Solobacterium*) (See Additional file 15: Table S13). Via deaminase enzyme activities, amino groups are removed from larger molecules and ammonia is formed as a byproduct. The alkaline ammonia from these activities may have an impact on the local pH, however this has not yet been experimentally verified. The upexpression of these enzymes in low pH may therefore not only result in a more alkaline micro-environment but also an increased activity of metabolic pathways associated with primary metabolism (e.g. biosynthesis of uridine from cytidine via the enzymatic activity of the cytidine deaminase).

### Three major activity signatures across biofilm maturation stages

In the following sections we further explore in detail three different patterns of gene expression activities that were observed during biofilm establishment and maturation. These signatures are represented by bacterial genomes that either increased or decreased in overall gene expression activity, or that showed high activity initially, then decreased, followed by recovery at the end of the growth period (Fig. 1, Additional files 10 and 11: Tables S8 and S9).

#### Increasing gene expression activities paralleled with biofilm maturation

**Fig. 4:**
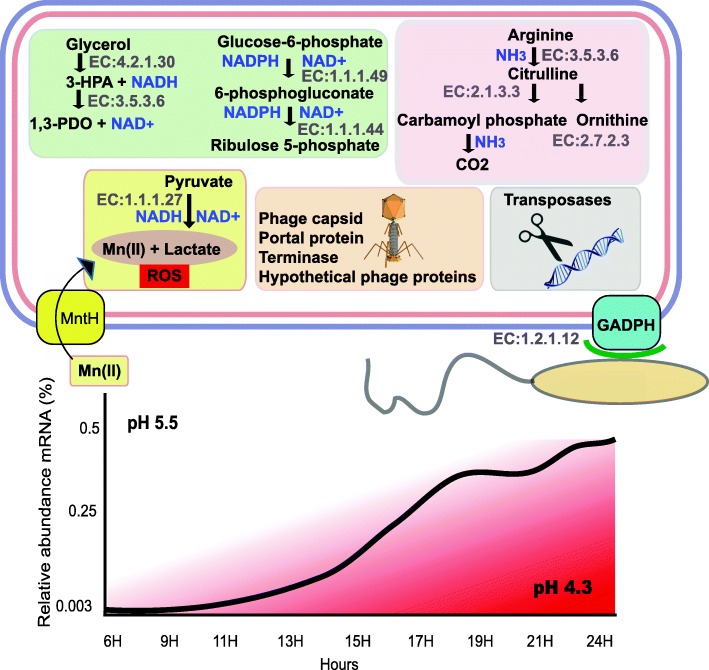
Metabolic functions represented by gene transcription activities that changed significantly in the *L. fermentum* genome. Y-axis: relative mRNA read abundance values in percent; x-axis: time points of biofilm maturation (x-axis). Upper panel: Metabolism of carbohydrates via the pentose pathway, pyruvate and glycerol oxidation, as well as the pH neutralizing arginine deiminase pathways were highly expressed during *L. fermentum*’s establishment as a secondary biofilm colonizer. Phage related proteins, and 67 transposases were also highly upexpressed suggesting that gene rearrangements and phage induction are of major importance in *L. fermentum* colonization success. Genes encoding two membrane proteins, a glyceraldehyde 3-phosphate dehydrogenase (GAPDH) and a manganese transporter (MntH) were also highly upexpressed and suggest that: *L. fermentum* is capable of forming a Lactate-Mn(II) complex that assists in the intracellular removal of reactive oxygen species (ROS); and employs GADPH in glycolysis or for attaching to primary colonizers in the in vitro biofilm community, which supports its further colonization and growth

In response to biofilm maturation, after six hours of growth when pH dropped from 5.5. to 4.7, 29 bacterial taxa showed an overall genome expression increase (> 1.5-fold) (See Additional files 10 and 11: Tables S8 and S9). Highly abundant taxa that showed these responses belonged to the *Veillonella* and *Streptococcus* genera (Fig. 1). When applying the MIDAS analysis pipeline, which uses marker genes to estimate relative abundance values of bacterial species overall, *S. parasangunis* showed the highest gene expression activity change (i.e. at 13 h of growth *S. parasangunis* contributed with 37% of the mRNA reads to the total community) (See Additional file 16: Table S14). Relative abundance estimates from our BWA mapping results also showed an increase in gene expression activity at this time point in less abundant taxa, i.e. bacteria contributing with less than 0.01% of the total community abundance (See Additional file 10: Table S8). Interestingly, the relative abundance of mRNA, representative of the known cariogenic bacterium *L. fermentum* increased from zero to 0.5% across the 24-h growth period (Fig. 4, See Additional file 16: Table S14). *L. fermentum* is commonly observed in advanced caries [52, 53], however to our knowledge, no information exists on the genes that are involved in supporting its colonization and expansion in a highly complex biofilm community. Here, by analyzing normalized read mapping data, obtained from the Open Reading Frame (ORF) database [26], we were able to predict *L. fermentum*’s most important gene transcription activities during niche expansion within the in vitro biofilm community (Fig. 4, See Additional file 3: Table S1). A drastic increase in gene expression (a 1000-fold upexpression) of the well-studied Arginine Deiminase (ADS) pathway (e.g. genes encoding: arginine deiminase EC: 3.5.3.6, ornithine carbamoyltransferase EC:2.1.3.3, carbamate kinase EC:2.7.2.3) was identified between nine hours to 24 h of growth, suggesting a high pH neutralization activity (Fig. 4, See Additional file 3: Table S1). Also, a glucose-6-phosphate 1-dehydrogenase and a 6-phosphogluconate dehydrogenase were highly upexpressed (51- and-70-fold, respectively) between nine hours and 24 h of growth, showing that the pentose phosphate pathway was of major importance for *L. fermentum* growth (Fig. 4, See Additional file 3: Table S1). In addition, a glyceraldehyde 3-phosphate dehydrogenase (GAPDH) was upexpressed 44-fold (DESeq values ranged between 7 and 4805). The function of this enzyme in lactobacilli was previously identified as an adhesion surface protein that plays important roles in binding to intestinal epithelial cells [54], and to bacterial cell surfaces [55]. However, it is also possible that GADPH was active in its most well-known cytoplasmic form, and was upexpressed due to glycolysis activities. It was notable that when GAPDH expression first increased (16-fold), at nine hours of biofilm maturation, *L. fermentum* was only a minor part of the community. At this time point GAPDH was one of few genes that showed high expression (See Additional file 3: Table S1). Another gene, also associated with attachment of lactobacilli to other bacteria, i.e. an autoinducer-2 like gene, was also upexpressed at this time point (18-fold) [56]. Our results suggest that association via attachment of *L. fermentum* cells with primary biofilm colonizers promotes an overall increase in *L. fermentum* gene expression activity. Other genes that are commonly associated with biofilm formation (e.g. genes encoding exopolysaccharides) were also expressed but at much lower levels (DESeq values ranged between 5 and 30). We also observed significant upexpression of genes encoding a manganese (Mn) transporting protein (MntH) (159-fold increase); a 1,3-propanediol (1,3-PDO) dehydrogenase (1000-fold); a phage portal and phage capsid protein (20- and 26-fold, respectively); and 67 transposase encoding regions (3 to 700-fold increase) (Fig. 4, See Additional file 3: Table S1). Lactobacilli are known to import and store Mn (II) intracellularly in response to reactive oxygen species (ROS) [57, 58]. In lactobacilli Mn (II) serves as a co-factor for superoxide dismutase (sodA), which catalyzes the degradation of toxic superoxide anion radicals. In addition, previous studies show that Mn(II) forms a complex with lactate inside the cells, which can directly catalyze the detoxification of O_2_^-.^[57–59] Due to the fact that we could not identify a *sodA* gene in the completed *L. fermentum* IFO 3956 genome, but we observed a drastic upregulation of the Mn-transport protein in concert with gene expression of the lactate dehydrogenase encoding genes (See Additional file 3: Table S1), it is likely that intracellular ROS was removed efficiently by the latter pathway, and contributed to *L. fermentum* establishment and growth (Fig. 4). Also, several genes encoding enzymes that are involved in NAD^+^ recycling were highly upexpressed at nine hours of biofilm maturation, which correspond to major important energy recycling mechanisms, which also support growth (Fig. 4). Taken together, the results here suggest that *L. fermentum* colonization and growth establishment in a highly acidic, oral biofilm environment is influenced not only by carbohydrate availability (e.g. glucose-6-phosphate, pyruvate), but also its capacity for ROS detoxification, NAD^+^ recycling, and pH neutralization by the ADS pathway.

Another highly active bacterium at nine hours of biofilm maturation was *S. parasangunis*, which is considered a commensal Gram-positive bacterium, and a primary colonizer of the human oral cavity involved in early development of dental plaque. Several oral *S. parasangunis* strains are also regarded as the primary pathogens involved in infective endocarditis [60]. They have been identified both in necrotic tissue and blood associated with infected heart valves [61–64] and not much is known about its activity in a complex community or as a low pH active species. Some of the most apparent functions carried out by *S. parasangunis* in the oral in vitro biofilm community were discussed previously, such as H_2_O_2_ production and pH neutralization. However, other less studied functions that were highly upexpressed after nine hours of biofilm maturation represented those involved in surface and cell attachment. For example, a total of 16 genes encoding LPXTG-domains were constitutively expressed at all time points after six hours of maturation (See Additional file 14: Table S12). 12 of these regions were upexpressed 2- to 15- fold as pH dropped below 5.5. Three of these domains were highly expressed with DESeq values ranging between ~ 2000 and 50,000 (See Additional file 14: Table S12). Earlier studies show that LPTXG-tagged proteins promote a multitude of interactions ranging from bacteria-to-bacteria interaction [65] providing ingenious strategies for evading the host’s immune response [66]. Overall, in this study, we identified 119 transcribed LPXTG-domains in 18 bacterial genomes, and the *S. parasangunis* genome was clearly in the lead for expressing genes with these domains (See Additional file 14: Table S12). The role of LPTXG proteins are highly underexplored in oral biofilm establishment and maturation, however our data suggests they have important roles in biofilm formation and should therefore be addressed further. Other *S. parasangunis*-genes associated with attachment, and that were constitutively expressed after nine hours of biofilm maturation, were genes encoding a fibronectin binding protein domain, and a *lemA* encoding gene (See Additional file 4: Table S2). *LemA* is a widely conserved two-component regulatory system that has been identified as regulating virulence factors and toxin production in *Pseudomonas syringae* [67]. The role of *lemA* in oral biofilm maturation and in interactions between *S. parasangunis* and other bacterial community members is not known, but due to the high gene expression activity that we observed here, *lemA* and its gene product is likely playing a significant role in biofilm maturation.

**Fig. 5:**
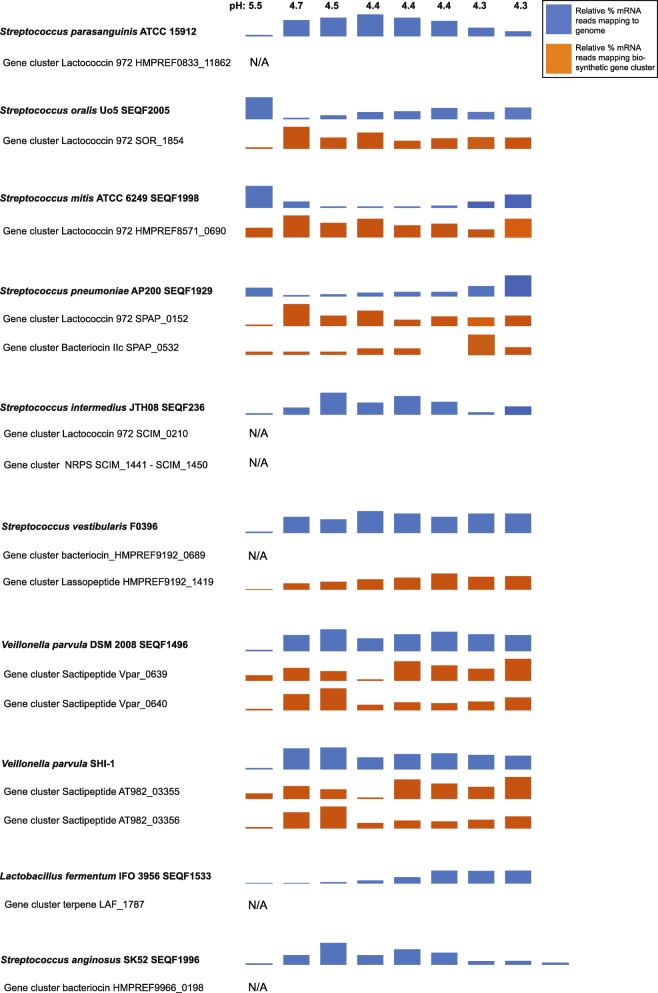
Gene transcription activity of biosynthetic gene clusters (BGCs) for individual community members. BGCs were identified by using the antiSMASH program available at https://antismash.secondarymetabolites.org/. Bars show relative abundance of mRNA reads that mapped to core-biosynthetic genes within each cluster. Relative transcription activity for individual genomes is shown as blue bars next to genome identifier while activity for BGCs is shown as orange bars next to the gene cluster name. X-axis shows different time points and pH stages. Five genomes harbor two BGCs each while five harbor one BGC. BGCs that show no expression are indicated with N/A

After six hours of growth, overall *S. parasangunis* gene transcription activity changed and a total of 1204 genes were significantly upregulated (fdr corrected *p*-values < 0.05) while 95 genes were significantly down regulated, indicating a stimulated metabolic activity (See Additional file 4: Table S2). A marked increase in sucrose catabolism was observed as soon as pH dropped below 5.5 (after nine hours of incubation) (See Additional file 4: Table S2). Genes encoding enzymes involved in the conversion of sucrose to fructose (EC:3.2.1.26), glucose (EC:5.4.2.2, EC:2.4.1.7), amylose (EC:2.7.7.27, EC:2.4.1.21), starch (EC:2.4.1.18) and cellobiose (extracellular) (EC:3.2.1.86) and glycogen (EC:2.4.1.18) were significantly upregulated (fdr corrected *p*-value < 0.005, fold change values ranged between 2.3 and 52) and then constitutively expressed throughout the incubation period (See Additional file 4: Table S2). Clearly, the availability of sugar stimulated metabolism and growth of *S. parasangunis* at all stages of biofilm succession. The shikimate pathway was highly upregulated at pH 4.7, indicating that *S. parasangunis* increased its synthesis of aromatic amino acids (tyrosine, phenylalanine and tryptophan) by using pyruvate from glycolysis and the pentose phosphate pathway (See Additional file 4: Table S2). These amino acids can then be further channeled into the biosynthesis of small molecules such as bacteriocins, which are known for their antibacterial activity. Interestingly, in parallel with the upregulation of the shikimate pathway, a bacteriocin-like pathway that harbors biosynthetic genes similar to the lactococcin 972 gene cluster [68] was also upregulated in the *S. parasangunis* genome (Fig. 5, See Additional file 4: Table S2). Together the results indicate a possible defense mechanism for *S. parasangunis* to outcompete other community members; allowing this bacterium to maintain its niche and high gene transcription activity [69]. Gene expression of biosynthetic gene clusters of individual oral biofilm community members is discussed in depth in a later section. Based on our observations here, showing that *S. parasangunis* has a very broad repertoire of active enzymes responsible for a variety of colonization and survival strategies, we suggest it has the potential to become one of the most competitive bacterial community members in oral biofilms. The role of *S. parasangunis* as an oral pathogen is unknown but several genes that were highly upexpressed in this study indicate the potential to damage human cells, but likely to be primarily for outcompeting other bacterial community members by carbon source competition, and the release of antibacterials. Its assumed role as an oral commensal bacterial community member, especially in a low pH, anaerobic and carbohydrate rich environment should be further investigated.

Several genomes belonging to the *Veillonella* genus also showed an overall upexpression of major genes at nine hours of growth (See Additional file 5: Table S3). The role of *Veillonella* in oral microbial ecology is gaining more interest as intriguing co-metabolic and signaling interactions have recently been discovered at the gene and molecular level in relationships with both commensal and pathogenic oral bacterial taxa [70, 71]. In our previous study we showed for the first time that *Veillonella* bacteria are highly active at low pH while within complex biofilm communities [26]. Here, at nine hours of biofilm maturation, a *V. parvula* DSM 2008 was highly active and showed upexpression of several Malate/L-lactate dehydrogenase genes (13-fold) (See Additional file 15: Table S12), indicating its importance in organic acid consumption at low pH (reference). In parallel, several iron/sulfur cycling encoding genes were upexpressed, e.g. a succinate dehydrogenase and fumarate reductase iron-sulfur protein (11-fold), an S-layer domain protein (29-fold), an iron-sulfur cluster binding protein (5-fold), and a cysteine desulfurase *SufS* subfamily (4-fold). Several *Veillonella* genomes showed upexpression (up to 44-fold) of *feoB* genes, which encode a ferrous iron (Fe^2+^) transport protein (See Additional file 5: Table S3). Fe^2+^ is the most abundant iron species under anaerobic conditions at low pH, and has also been implicated in iron-mediated biofilm formation [72]. Based on these findings, we propose that iron acquisition, which have also been described as important metabolic activity defining disease [73], is an important phenotypic trait that support establishment and growth of *Veillonella* in oral biofilms [74].

### Decreasing gene expression activity paralleled with biofilm growth

A total of nine genomes showed a relative decrease of gene expression (1.5-fold down expression or more) at nine hours of growth, at pH 4.7 (See Additional file 11: Table S9). These genomes remained active throughout the growth period, however overall gene expression was significantly lower. When applying the abundance estimates from the DESeq normalized data, the ranges of relative mRNA read contribution of these genomes ranged between 0.009% to 13% to the total community abundance (See Additional file 10: Table S8). A *Streptococcus* oral taxon 070 F0441 showed the highest abundance, 12.8% at six hours of growth, and decreased in abundance to 8.6% at nine hours. The most drastic decrease (10-fold) was observed for *Klebsiella pneumoniae* Kp342, and *S. pyogenes* MGAS10270 (See Additional file 11: Table S9). Other taxa that showed decreased gene expression (1.5-fold down expression or more) were *L. salivarius* UCC118, *S. mitis* ATCC 6249*, S mitis* biovar 2*, Streptococcus* oral taxon 058*,* and *Streptococcus* oral taxon 058, *G. elegans* ATCC 700633. The *Klebsiella pneumoniae* genome that showed a 10-fold decrease at nine hours, downexpressed over 2066 genes, which represented most of its genes that had mapping reads in this study (See Additional file 8: Table S6). Genes that supported *Klebsiella pneumoniae* growth at the previous growth stage (six hours) were related to those encoding DNA replication proteins, lipoproteins, ureases, amino acid-, sugar- and iron-transporters, lactate dehydrogenase, outer membrane proteins (e.g. OmpA), and peptidases. Due to the rapid switch of *Klebsiella pneumoniae* gene activity below the pH of 5.5, we propose that low pH has a major influence of *Klebsiella pneumoniae* gene expression activity and growth (See Additional file 8: Table S6).

### Community members whose genome expression activities recover at later stages of biofilm growth

*S. infantis* showed an interesting increase in overall genome transcription across time, which started with a 5.5% mRNA contribution to the total community relative read abundance (six hours) and at the end of the study (24 h), it increased to 31.2% (Fig. 1, See Additional files 17: Table S14). *S. infantis* is generally regarded as a commensal community member, which a previous co-cultivation study identified it as a key mediator in pathogen invasion resistance [2]. This was shown in a co-culture of three oral bacterial species; *S. saprophyticus*, *S. infantis* and *S. sanguinis* [2]. In the absence of a foreign invader (i.e. *E. coli*), *S. infantis* represses *S. sanguinis*’ capacity to produce H_2_O_2_, resulting in minimal H_2_O_2_ levels within the community. When encountering *E. coli*, *S. saprophyticus* acts as a sensor due to its ability to detect the presence of *E. coli,* which then initiates the invasion resistance response by producing diffusible signals. These signals are thought to relay the information to *S. infantis*, which not only alleviates its suppression on H_2_O_2_ production in *S. infantis* but also stimulates the *S. sanguinis* H_2_O_2_-producing capability. Interestingly, the resulting increased H_2_O_2_ levels were observed to exert an inhibitory effect on the invading *E. coli*. In our study, gene transcription data suggest that H_2_O_2_ was produced at all growth stages by various *Streptococcus* species, including *S. infantis*, by the activity of SOD, *spxB* and *lctO* encoding genes (See Additional files 6 and 14: Tables S4 and S12), as discussed in previous section. *S. infantis* specific role in signaling via H_2_O_2_ could not be revealed here due to the complexity of the studied community. However, a marked gene transcription signal of H_2_O_2_ production was detected, represented by a broad diversity of community members (e.g. *Veillonella*, *Streptococcus, Actinomyces, Granulicatella*), including *S. infantis*, showing a strong defense against pathogen invasion. In the *S. infantis* genome, several genes that were significantly upregulated at nine hours of biofilm maturation, after pH had dropped below 5.5, were related to purine metabolism and cell division (e.g. a *xerS* gene, ~ 18-fold; a phosphoribosylaminoimidazolesuccinocarboxamide synthase gene, 43-fold) (See Additional file 6: Table S4). At the last growth stage, the taxonomic signatures responsible for overall community gene transcription had changed dramatically and S*. infantis* was one of the most active community members (i.e. its contribution was ~ 11.6% to the total mRNA community abundance) (See Additional file 10: Table S8). S*. infantis* gene expression was represented by upexpression of a *nagZ*-like gene (2-fold), an *EndoE*-like mannosyl-glycoproteinendo-beta-N-acetylglucosaminidase encoding gene (13-fold), and a Gram-positive signaling peptide (3-fold) (See Additional file 6: Table S4). Previous studies suggest that these genes are involved in peptidoglycan hydrolysis, biofilm dispersal and autolysis [75–77]. Taken together, our study suggests that *S. infantis* becomes a highly competitive community member as biofilm maturation proceeds, due to its capacity to thrive in low pH and metabolizing amino acids, likely derived from hydrolysis of cell wall components (perhaps from dead cells), via NagZ and EndoE enzymatic activities.

*G. adiacens* showed a similar gene transcription response as *S. infantis* (i.e. an increase in overall transcription activities at the latest growth stages). Members belonging to the *Granulicatella* genus are considered normal component of dental plaque, and were originally known as ‘nutritionally variant streptococci’. However, they have also been associated with a variety of invasive oral infections (e.g. endodontic infection, dental abscesses), and are noted as a cause of bacterial endocarditis [78]. To our knowledge, gene transcription behaviors of *G. adiacens* in oral biofilms have not been studies this far, but here we could confirm that *G. adiacens* is an early colonizer and that its overall gene transcription activity is highly dynamic in response to biofilm maturation and pH stress. At six hours of growth, *G. adiacens* contributed with 5.1% of mRNA reads to the total community, but at later growth stages (after nine hours of growth), its activity dropped (e.g. relative mRNA read abundance was only 0.98% at 13 h of growth) (See Additional file 16: Table S14). However, at 24 h of growth when pH was 4.3, *G. adiacens* regained its activity and contributed to 4.1% of the total mRNA reads (See Additional file 16: Table S14). At this time point, several virulence related mechanisms were identified such as the upregulation of peptidases (See Additional file 7: Table S5). A dipeptidase belonging to the C69 family was highly upexpressed (32-fold) at a pH of 4.3 (See Additional file 7: Table S5). Interestingly, a previous study has shown that C69 is a cysteine peptidase, known for being highly capable of damaging human tissues in acidic pH [79]. In the biofilm community it is likely C69’s role is involved with protein hydrolysis of cell wall material from dead bacterial cells that had accumulated in the community over time. Other genes associated with cell-wall recycling were also upexpressed, e.g. a LD-carboxypeptidase (5-fold), N-acetylmuramic acid 6-phosphate etherase (five-fold), UDP-N-acetylglucosamine 1-carboxyvinyltransferase (five-fold), anhydro-N-acetylmuramic acid kinase (six-fold). *G. adiacens* upexpressed a gene encoding a bacterial cell surface capsule synthesis protein, which may explain an important mechanism to protect its cells from acid stress (See Additional file 7: Table S5). A pheromone encoding gene *camS* was also upexpressed (six-fold) at the latest growth stage (See Additional file 8: Table S6). CamS is an ortholog of the Putative pheromone cAM373 precursor, which is present in the genomes of many *Bacilli*, *Listeria*, *Thermoanaerobacter* and lactobacilli [80]. Although it is not known whether the CamS-peptides produced by different genera actually represent pheromones significant to these organisms, it is possible that they could play a role in the acquisition of plasmids contributing to cross-species spread of genes. Taken together, the dynamic gene expression pattern we observe for *G. adiacens* indicates it has the capacity to stay both metabolically activity and social with other community members during a broad range of pH and biofilm maturation stages. Similar to *S. infantis*, *G. adiacens* seems to derive both carbon and energy sources by protein hydrolysis of cell wall components.

### Expression of biosynthetic gene clusters in the complex biofilm community

Diffusible and microbe-associated small molecules (SM) often play important roles in mediating intra- and/or interspecies interactions in complex ecological systems [81–84]. Several recent studies have begun to examine the human microbiome for identifying small molecules as well as scrutinizing their biological roles. For instance, the nonribosomal peptide-polyketide (NRP-PK) hybrid metabolite colibactin, derived from enteric *E.coli* was found as a genotoxic SM contributing to colon cancer [85, 86]. Similarly, the *Staphylococcus aureus* NRP product aureusimines were identified to induce bacterial virulence [87], whereas polysaccharide A from *Bacteroides fragilis* has been shown to modulate the gut mucosal immune response [88, 89]. Most recently, the thiopeptide antibiotic lactocillin from a vaginal isolate of *Lactobacillus gasseri* was characterized and showed a potential role in protecting the vaginal microbiota against pathogen invasion [90]. Another recent study discovered the novel NRP antibiotic lugdunin from the nasal commensal *Staphylococcus lugdunensis* strains, which exhibited the ability to prevent nasal staphylococcal infections [91]. In this study, biosynthetic gene clusters (BSCs) encoding SMs were searched in 31 of the most active genomes (See Additional file 10: Table S8) within the community by using the antiSMASH v. 3.0 pipeline [34], which resulted in the identification of 130 BGCs (See Additional file 17: Table S15). Most antiSMASH-annotations represented saccharides (e.g. capsular polysaccharides), fatty acids or putative pathways, and only 15 belonged to known compound classes, such as bacteriocin IIc, lantipeptides, sactipeptides, non-ribosomal peptides, lassopeptides and terpenes (See Additional file 17: Table S15). The latter groups were detected in 10 genomes. Eight genomes (e.g. *Streptococcus* sp. oral taxon 058, *Streptococcus* sp. GMD4S, *S. tigurinus* 1366, *S. peroris* ATCC 700780, *V. atypica* ACS-134-V-Col7a, *Veillonella* sp. ACP1, *G. adiacens* ATCC 49715) did not harbor any BGCs suggesting that these species employ other signaling pathways and molecules to interact with the rest of the community. By performing mRNA-read mapping to the 15 identified gene clusters we characterized nine that were differentially expressed across pH stages while six were not expressed at all (Fig. 5). One predicted gene cluster encoding a putative lactococcin 972 molecule, a bacteriocin with a narrow growth inhibition spectrum [92], was upexpressed in genomes that decreased in overall activity at the lower pH stages (i.e. *S. oralis* Uo5, *S. mitis* ATCC6249, *S. pneumoniae* AP200), while the cluster remained completely silent in genomes that increased in transcription activity as pH decreased (*S. parasangunis* ATCC15912 and *S. intermedius* JTH08) (Fig. 5). A previous study show that Lactococcin 972 is a nonlantibiotic bacteriocin that controls growth by inhibiting septum biosynthesis in *Lactococcus lactis* (formerly classified as *Streptococcus*), which also could be its role here. Based on these findings we suggest that the production of Lactococcin 972 may reflect a strategy to outcompete other oral community members under pH stress, allowing the producer to occupy some niche space and wait around until more favorable growth conditions appear. In addition, the Lactococcin 972-like gene cluster seems to be relatively broadly distributed within the *Streptococcus* genus as it was identified in five genomes representing different species (See Additional file 17: Table S15).

Another key finding was the presence of a gene cluster in the genome of *V. parvula* DSM 2008 and *V. parvula* SHI-1 [93], which encode functions involved in the biosynthesis of a putative sactipeptide molecule (See Additional file 18: Figure S2, See Additional file 17: Table S15). The basic gene cluster core consists of a Radical SAM enzyme and several cysteine residues [94, 95], with the latter contributing to thioether bond formation of sactipeptides. Earlier studies show that sactipeptides are harbored by species within the *Bacillus* and *Clostridium* genera [94, 95]. Here for the first time, we identified a sactipeptide-like gene cluster associated with the *Veillonella* genus. Biosynthetic genes within the clusters were upexpressed at pH below 5.5, both for the *V. parvula* DSM 2008 and the *V. parvula* SHI-1 genomes; coinciding with their overall increased transcription activity (Fig. 5). Based on these finding we suggests that sactipeptides may contribute to *V. parvula*’s competitive success in low pH and perhaps it is also involved in interaction with *Streptococcus* species, which have been confirmed in earlier studies [96]. Their biological function is for the most part unknown, yet functions such as cannibalism inducing factor (i.e. sporulation killing factor), which causes cell-lysis and delay of sporulation [97] as well as antimicrobial activities against various Gram-positive and Gram-negative bacteria [98] have previously been reported.

A putative lasso peptide encoding gene cluster was also identified in *S. vestibularis* F0396, which also was transcribed at all growth stages but to a higher extent after six hours of growth when pH dropped below 5.5 (See Additional file 18: Table S15). Lasso peptides are a class of bioactive ribosomally synthesized and post-translationally modified peptides (RiPPs) [99], which have a wide range of interesting biological activities including antimicrobial, enzyme inhibitory, and receptor antagonistic activities [100]. Based on our findings it is possible that *S. vestibularis* employs this gene cluster to produce an antibacterial compound that gives this bacterium a competitive advantage in the oral biofilm community.

The role of secondary metabolites in oral biofilms virulence is largely unexplored however, here we provide new insights into which molecules may be of importance in oral microbial ecology during biofilm establishment and maturation. Importantly, most of antiSMASH-predicted BGCs that we identified are not discussed here due to that no knowledge exists on their structures and activities. A major future challenge is to isolate, characterize and experimentally validate these molecules to gain a deeper knowledge of their roles in oral microbial interactions and their eventual role in health and disease.

## Conclusions

Most of our current knowledge of the complex human microbiome is derived from 16S rRNA gene sequencing and metagenomics techniques that can determine the presence and absence of microbes and quantify genes. In this study by applying a metatranscriptomic approach, which targets mRNA synthesized by active bacterial genes and genomes, we were able to obtain a deeper understanding of bacterial behaviors and ecosystem functions as a complex oral biofilm community initially assembles and matures. Combining whole community shotgun sequencing approach of DNA with mRNA metatranscriptomics allowed us to quantify the highly dynamic functional responses and species-unique gene expression patterns related to biofilm community invasion resistance, cell-to-cell signaling, cell attachment mechanisms, iron sequestration, low pH stress responses, and unique secondary metabolite biosynthetic pathways throughout 24 h of growth. Moreover, we fully characterized the temporal gene expression and discovered previously unknown functions that were associated with the cariogenic pathogen *Lactobacillus fermentum* during niche expansion at low pH that could help to understand its high abundance and prevalence in severe caries disease.

## Additional files

Additional file 1: Supplementary Information Additional file 19. (DOCX 207 kb)

Additional file 2: **Figure S1.** Distribution of key bacterial taxa in the biofilm community obtained by DNA and mRNA deep sequencing approaches, respectively. Heat maps show relative abundance estimations of DNA, and mRNA reads representing the most abundant reference genomes. DNA read mapping was performed for all stages of growth (0 to 24 h, pH 7.2 to 4.3). mRNA read mapping was performed with libraries representative of 6–24 h of growth (pH 5.5 to 4.3). Two replicate DNA libraries were prepared from each growth stage, while three replicate mRNA libraries were prepared, except from six hours, 21 h, and 24 h of growth, for which two libraries were prepared. Metagenomics (MG) and metatranscriptomics (MT) read mapping results are shown for the most abundant *Streptococcus* species in panel A while other bacterial taxa are presented in panel B. (PDF 1603 kb)

Additional file 3: **Table S1.** DESeq normalized RNASeq data representing the *Lactobacillus fermentum* IFO 3956 genome in the in vitro biofilm model system across different pH stages. Average DESeq values are represented for all pH stages. (XLS 473 kb)

Additional file 4: **Table S2.** DESeq normalized RNASeq data representing expressed *Streptococcus parasangunis* ATCC15912 genes. Average DESeq values are represented for all pH stages. (XLS 1186 kb)

Additional file 5: **Table S3.** DESeq normalized RNASeq data representing eight different *Veillonella* genomes in the in vitro *b*iofilm model system across different pH stages. Average DESeq values are represented for all pH stages. (XLS 3776 kb)

Additional file 6: **Table S4.** DESeq normalized RNASeq data representing the *Streptococcus infantis X* SEQF2216 genome in the in vitro biofilm model system across different pH stages. Average DESeq values are represented for all pH stages. (XLS 575 kb)

Additional file 7: **Table S5.** DESeq normalized RNASeq data representing the *Granulicatella adiacens* ATCC 49175 genome in the in vitro biofilm model system across different pH stages. Average DESeq values are represented for all pH stages. (XLS 605 kb)

Additional file 8: **Table S6.** DESeq normalized RNASeq data representing the *Klebsiella pneumoniae* 342 genome in the in vitro biofilm model system across different pH stages. Average DESeq values are represented for all pH stages. (XLS 893 kb)

Additional file 9: **Table S7.** Sequence reads before and after quality filtering, and mapping results for all metagenome (MG) and metatranscriptome (MT) libraries. (XLSX 17 kb)

Additional file 10: **Table S8.** Read mapping results showing DNA and mRNA read mapping reproducibility between replicate libraries. Read mapping was performed using reference genomes representative of oral bacteria (see Methods section). A separate spreadsheet is provided for relative abundance values of mRNA reads mapping to oral reference genomes. Fold change calculations are included in this sheet showing differences between: 9 h and 6 h of growth; 11 h and 9 h of growth; and 24 h and 6 h of growth. (XLSX 161 kb)

Additional file 11: **Table S9.** Relative abundance (RA) and fold changes values representing mRNA reads mapping to bacterial genomes, and that changes (increased or decreased) 1.5-fold at 9 h of biofilm maturation, when pH dropped below 5.5. RA for each genome was calculated from DESeq normalized metatranscriptomics reads. (XLSX 44 kb)

Additional file 12: **Table S10.** Linear regression analysis between cell division genes (*ftsA, ftsZ*) and single copy genes (Amphora set of 42 single copy genes). Analyses were conducted for *Lactobacillus fermentum* IFO3956 (Lf) and *S. parasangunis* ATCC15912 (Spa) from normalized metagenomic read mapping data. (XLSX 62 kb)

Additional file 13: **Table S11.** Ratio estimates of metatranscriptome (MT) and metagenome (MG) read mapping results for individual genomes. Values are based on relative abundance estimates from mRNA or DNA read mapping. MT abundance values were divided with MG abundance values. High ratios suggest that genomes are highly active in the community. (XLSX 63 kb)

Additional file 14: **Table S12.** DESeq normalized RNASeq data representing specific functions associated with virulence in the in vitro biofilm community. Gene expression of seven groups of virulence functions were analyzed across pH stages (i.e. LPXTG-motifs, *feoB* genes, capsular polysaccharide genes, Cpl protease encoding genes, *lemA* genes, oxygen radical associated genes, methionine-R-sulfoxide reductase genes). (XLSX 129 kb)

Additional file 15: **Table S13.** DESeq normalized RNASeq data representing expressed genes associated with ammonia production and pH neutralization. (XLSX 123 kb)

Additional file 16: **Table S14.** Relative abundance values of metagenomics and metatranscriptomics reads calculated by using the Metagenomic Intra-Species Diversity Analysis System (MIDAS) pipeline. Bacterial taxa that contributed with 0.5% or more to the total DNA or mRNA read abundance at any time point were included in the analysis. (XLSX 51 kb)

Additional file 17: **Table S15.** Gene expression data of putative biosynthetic gene clusters (BSCs) identified in the 31 most active bacterial genomes in the in vitro biofilm community by antiSMASH v. 3.0. A total of 130 biosynthetic BSCs were identified, however only nine were transcriptionally active in the biofilm community. Genomes with no BGC are presented at the end of the table. (XLSX 52 kb)

Additional file 18: **Figure S2.** A putative sactipeptide biosynthetic gene cluster that was identified in *Veillonella parvula* genomes. The gene cluster was identified by using the antiSMASH program available at https://antismash.secondarymetabolites.org/. It was actively transcribed between six hours and 24 h of growth when pH dropped below 5.5 in both genomes. Genes are color coded based on putative function. The six-cysteine peptide SCIFF and Radical SAM motifs represent core modules of this group of natural products. (PDF 102 kb)

Additional file 19: **Table S16.** DESeq calculations showing differential gene expression of annotated open reading frames (ORFs) between time points and pH stages. Genes that were significantly changing in expression (fdr corrected *p*-value < 0.05) are also presented in separate spreadsheets for each comparison. (XLSX 48991 kb)

## Abbreviations

16S rRNA: A component of the 30S small subunit of a prokaryotic ribosome that binds to the Shine-Dalgarno sequence
ADS: Arginine deiminase pathway
BGC: Biosynthetic gene cluster
CamS: A pheromone precursor
DESeq: A computational package that can analyze count data from high-throughput sequencing assays
DNA: Deoxyribonucleic acid
Fe^2+^: Iron ion
GAPDH: Glyceraldehyde 3-phosphate dehydrogenase enzyme
GC-MS: Gas chromatography-mass spectrometry
GTP binding protein: Guanine nucleotide-binding proteins
H_2_O_2_: Hydrogen peroxide
LC-MS: Liquid chromatography-mass spectrometry
LPXTG-domains: A protein sorting signal where X could be any amino acid
MG: Metagenomics
Mn: Manganese
mRNA: Messenger ribonucleic acid
MT: Metatranscriptomics
NAD^+^: Nicotinamide adenine dinucleotide ion
NRP-PK: Non ribosomal peptide- polyketide
O_2_^−^: Free radical oxygen species
O_2_: Oxygen
ORF: Open reading frame
OTU: Operational Taxonomic Unit
PSM: Peptidic small molecule
RNA: Ribonucleic acid
ROS: Reactive oxygen species
SM: Small molecule
SOD: Superoxide dismutase enzyme
