# Supplementary material for: Uncovering complex microbiome activities via metatranscriptomics during 24 hours of oral biofilm assembly and maturation
Source: Microbiome. 2018 Dec 6;6:217. doi: 10.1186/s40168-018-0591-4 (PMC6284299; doi:10.1186/s40168-018-0591-4)
Supplement: Supplementary file 1 — Supplementary Information Additional file 19. (DOCX 207 kb) [file 40168_2018_591_MOESM1_ESM.docx]

**Additional files - Supplementary Information**

Supplementary Methods

*SHI growth medium, growth-well preparation and incubation conditions*

SHI medium[1] (1 L) was prepared as follows: 10 g protease peptone (Thermo Fisher Scientific, Waltham, MA), 5 g trypticase peptone (BD Bacto, Radnor, PA), 5 g yeast extract (BD Bacto), 2.5 g KCl, 10 ml hemin solution (from 50 mg hemin stock, Millipore Sigma, Billerica, MA), 0.06 g urea, 0.17 g arginine, 2.5 g mucin (type III, porcine, gastric, Millipore Sigma), 990 mL distilled water. This solution was autoclaved in 121°C for 15 min, cool to 50°C: 1 mg vitamin K (Alpha Aesar, Ward Hill, MA) dissolved in water and filter sterilized 50 mL sheep blood (CS1122, Colorado serum company, Denver, CO), I mL NAM solution was added after cooling. NAM stock solution is prepared as follows: 100 mg N-Acetyl Muramic Acid (Millipore Sigma) was dissolved in 10 mL distilled water. The stock solution was then filter sterilized and stored in -20°C. Cell-free saliva for coating growth-well surfaces was obtained by centrifugation of the pooled saliva at 2,600 g for 10 min. 200 μL of the cell-free top layer was withdrawn with a sterile pipette and used to coat each bottom surface in a 24-well plate by pipetting saliva up and down until bottom surfaces were completely covered. The plate was dried in 37°C for 30 minutes and moved to a flow hood UV- chamber for 45 minutes to allow cross-linking of saliva proteins. Saliva samples were collected and pooled from six healthy subjects, age 25–35 years as described by Edlund and colleagues (2013)[2]. In each well, 10 μl of the pooled saliva was inoculated into 980 μl of SHI medium, and 10 μl of sucrose (2.7 mM final concentration).

*Sample collection, mRNA synthesis and sequencing of in vitro biofilms*

Samples for mRNA were collected at designated pH stages after removing 0.5 ml of the spent SHI medium and adding 2 volumes of RNAProtect (QIAGEN Sciences Inc. USA, Valencia, CA) to the remaining biofilm. mRNA was collected from duplicate (2) or triplicate (3) growth wells from 6 hrs to 24 hrs as follows (a total of 21 samples): 6 hrs at pH 5.5 (2), 9 hrs at pH 4.7 (3), 11 hrs at pH 4.5, 13 hrs at pH 4.4, 15 hrs at pH 4.4, 17 hrs at pH 4.4 (3), 21 hrs at pH 4.3 (2), 24 hrs at pH 4.3 (2). Too little mRNA was present for obtaining high quality sequencing libraries from 3 hours of growth at pH 6.5. No mRNA was obtained from the first time point (pH 7.2) as no growth had occurred (time point zero). Growth wells containing biofilms that were maintained in RNAProtect were pooled for a single sample, see mRNA extraction below, to represent one single mRNA library replicate. Biofilm samples were collected and pooled from separate growth wells, see below. Our previous study show that bacterial diversity is basically identical over a growth period of 48 hours between growth wells that were seeded in the same experiments using the same saliva pool[2]. Therefore, pooling biofilms for DNA and RNA extraction was justified here. The biofilm/RNAProtect-suspensions for each replicate were immediately mixed by vortexing for 5 s and incubated in room temperature for 5 min prior to centrifugation for 10 min at 5,000 x g following manufacturer’s recommendations. The pelleted biofilms were stored at -80°C for one week prior to RNA extraction. Each replicate (in total 21) was subjected to RNA extraction as follows: pelleted and frozen biofilms were thawed on ice in 350 ul lysis buffer (mirVana RNA extraction kit, Life Technologies, Carlsbad, CA). Each replicate was transferred to a bead beating tube (PowerBiofilm RNAisolation kit, MoBio Laboratories Inc., Carlsbad, CA) and inserted into a Mini-bead beater (Bio Spec Products, Bartlesville, OK). Samples were homogenized twice at speed setting ‘homogenize’ for 30 s. Samples were put on ice between and after beating events. Total RNA extraction and purification was performed by using the mirVana RNA extraction kit (Life Technologies) and the RNA Clean and Concentrator™ kit (Zymo Research, Irvine, CA), respectively. DNA was removed from the samples by adding 1 μl (2U) Turbo™ DNAse (Life Technologies) and incubation in 37°C for 30 min. After DNA removal, 16S rRNA PCR was performed by using the same protocol and primers as described by McLean and colleagues[3] (for 32 cycles of amplification to verify that DNA was removed). To remove rRNA in total RNA extracts the RiboZero™Magnetic Kit (Epicenter, Madison, WI) was employed according to manufacturer’s directions. mRNA was purified by using the Zymo RNA Clean and Concentrator™ kit (Zymo Research). RNA concentration and integrity was monitored before and after rRNA removal by using the Agilent RNA 6000 Nano Kit (Agilent Technologies, Inc. Santa Clara, CA) and the Agilent RNA 6000 Pico Kit (Agilent Technologies), respectively. After rRNA removal mRNA concentration ranged between 1.8 and 2.1 ng/μl in all samples. mRNA library from rRNA-depleted RNA was generated by using random-primed mRNA synthesis methods according to the ScriptSeq™v2 RNA-Seq Library Preparation Protocol (Epicenter). Prior to second strand mRNA synthesis the di-tagged mRNA was purified by using the Agencourt AMPure XP system (BeckmanCoulter, Carlsbad, CA). Index-reads supplied with the ScriptSeq Kit were added to the libraries, which then were PCR amplified for 15 cycles. RNA-Seq libraries were purified and quantified by using the Agencourt AMPure XP system (BeckmanCoulter) and the Agilent DNA 1000 protocol (Agilent Technologies), respectively. Sequencing of mRNA libraries was performed by using an Illumina NextSeq platform (150 bp paired end reads). Using each sample's individual barcodes, the Illumina data was deconvolved into the respective samples. After trimming the barcodes, low-quality and short sequences were removed by using the CLC ngs-cell program and Perl scripts using the following settings: -c quality score 20, -f phred quality score 33, -m minimum length of sequence to keep after filtering 55 bp. A Dust Masker program also was used to mask low complexity parts of the CLC-filtered reads. The Ribopicker program v. 0.4.3 [4]was used to remove 16S rRNA gene fragments.

### *DNA extraction and sequencing*

DNA was isolated using the DNeasy Blood and Tissue Kit (QIAGEN Sciences Inc. USA, Valencia, CA) and eluted in a final volume of 200 μl water. DNA was collected from two additional time points as described for mRNA collection above; zero hours of growth (start of experiment at pH 7.2), 3 hours at pH 6.5. DNA sequencing libraries were prepared using the Nextera XT DNA Library Preparation kit (Illumina Inc. La Jolla, CA). The yield of DNA was low in the zero and 3h samples (10 ng/ul), and 200 ng/ul in the remaining samples (6-24 h). 1 ng of DNA was used to prepare each Nextera XT library. Whole Genome Shotgun sequencing (wgs) was performed on total DNA extracted from duplicate biofilms growing in individual growth wells at 0, 3, 6, 9, 11, 13, 15, 17, 21 and 24 hrs of growth in SHI medium. A total of 22 libraries were prepared for wgs. Wgs sequences (150 bp fragment and paired-end reads) were obtained from the Illumina NextSeq platform, quality trimmed and filtered using CLC workbench software v. 6.0.1 (CLCbio, Aahus, Denmark). Low-quality and short sequences were removed as described above.

*Reproducibility of mRNA sequencing libraries at community transcription level*

The generation of high-quality mRNA reads from 21 RNA extracts, representing biological replicates was successful. Only 3% to 12% of the mRNA reads consisted of rRNA-encoding reads after using the RiboZero rRNA removal method (See Additional file 10: Table S7). For unknown reasons, one library from 6 hours of growth at pH 5.5 (i.e. 6HP2-PE-IL2-1_S2) contained 32% rRNA reads and therefore approximately 10M reads were removed from this library (See Additional file 10: Table S7). After quality trimming and rRNA read removal the number high-quality mRNA reads that remained in each library ranged between 18,555,628 to 32,712,842 (See Additional file 10: Table S7). DESeq normalized counts were calculated for the individual genomes based on results from Burrows-Wheeler Aligner (BWA) mapping.

*Generation of a non-redundant ORF dataset for functional annotation*

A total of 3,979,383 high-quality ORFs deriving from 1,521 bacterial genomes were initially selected for this dataset[5]. These ORFs were then combined with *de novo* assembled ORFs from our previously obtained metagenomic sequencing data representing a similar *in vitro* biofilm experiment[2] as well as *de novo* assemblies of the cDNA from the metatranscriptomic time point samples. The combined number of ORFs was 5,206,280. To reduce redundancy, ORFs were clustered at 100% identity (over 90% alignment coverage) using cd-hit-est[6] and only cluster representatives were used subsequently (referred to as “non-redundant” ORFs[5]. The clustering resulted in 2,288,459 non-redundant ORFs, and these were annotated by the JCVI prokaryotic metagenomics pipeline[5, 7]. All trimmed mRNA reads were mapped onto this ORF data set and paired reads restriction was enforced meaning that if paired reads could not be matched to the same ORF, both reads were excluded from the analyses (See Additional file 19: Table S16). After mapping the DESeq normalization protocol was conducted, which generated fold change values of gene expression for each matching gene within the ORF-dataset.

**References**

1. Tian Y, He X, Torralba M, Yooseph S, Nelson KE, Lux R, McLean JS, Yu G, Shi W (2010) Using DGGE profiling to develop a novel culture medium suitable for oral microbial communities. Mol Oral Microbiol 25: 357-367. doi: 10.1111/j.2041-1014.2010.00585.x

2. Edlund A, Yang Y, Hall AP, Guo L, Lux R, He X, Nelson KE, Nealson KH, Yooseph S, Shi W, McLean JS (2013) An in vitro biofilm model system maintaining a highly reproducible species and metabolic diversity approaching that of the human oral microbiome. Microbiome 1: 25. doi: 10.1186/2049-2618-1-25

3. McLean JS, Fansler SJ, Majors PD, McAteer K, Allen LZ, Shirtliff ME, Lux R, Shi W (2012) Identifying low pH active and lactate-utilizing taxa within oral microbiome communities from healthy children using stable isotope probing techniques. PLoS One 7: e32219. doi: 10.1371/journal.pone.0032219

4. Schmieder R, Lim YW, Edwards R (2012) Identification and removal of ribosomal RNA sequences from metatranscriptomes. Bioinformatics 28: 433-435. doi: 10.1093/bioinformatics/btr669

5. Edlund A, Yang Y, Yooseph S, Hall AP, Nguyen DD, Dorrestein PC, Nelson KE, He X, Lux R, Shi W, McLean JS (2015) Meta-omics uncover temporal regulation of pathways across oral microbiome genera during in vitro sugar metabolism. ISME J 9: 2605-2619. doi: 10.1038/ismej.2015.72

6. Li W, Godzik A (2006) Cd-hit: a fast program for clustering and comparing large sets of protein or nucleotide sequences. Bioinformatics 22: 1658-1659. doi: 10.1093/bioinformatics/btl158

7. Tanenbaum DM, Goll J, Murphy S, Kumar P, Zafar N, Thiagarajan M, Madupu R, Davidsen T, Kagan L, Kravitz S, Rusch DB, Yooseph S (2010) The JCVI standard operating procedure for annotating prokaryotic metagenomic shotgun sequencing data. Stand Genomic Sci 2: 229-237. doi: 10.4056/sigs.651139
