# Supplementary figures and images for: Uncovering complex microbiome activities via metatranscriptomics during 24 hours of oral biofilm assembly and maturation
Source: Microbiome. 2018 Dec 6;6:217. doi: 10.1186/s40168-018-0591-4 (PMC6284299; doi:10.1186/s40168-018-0591-4)

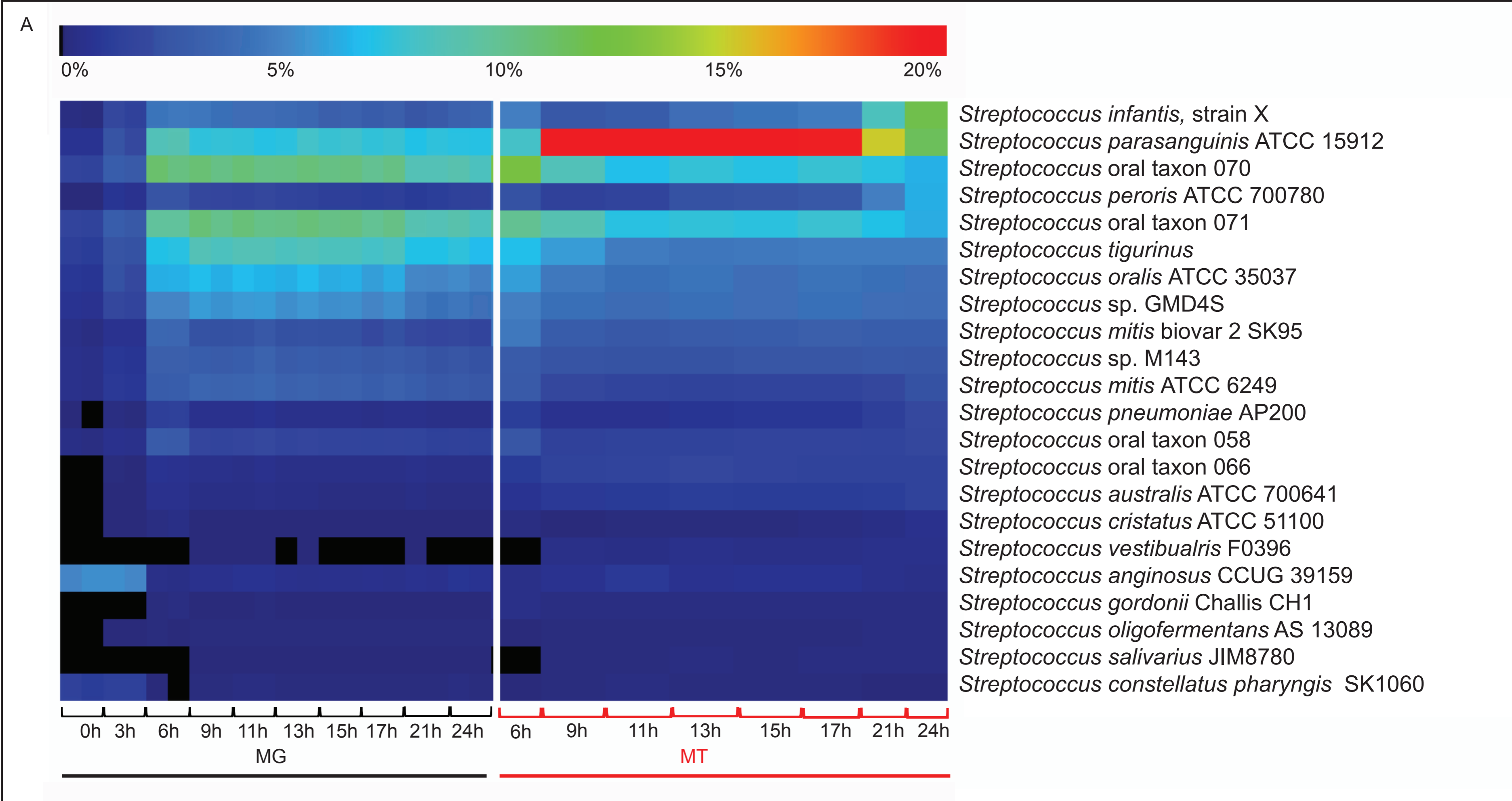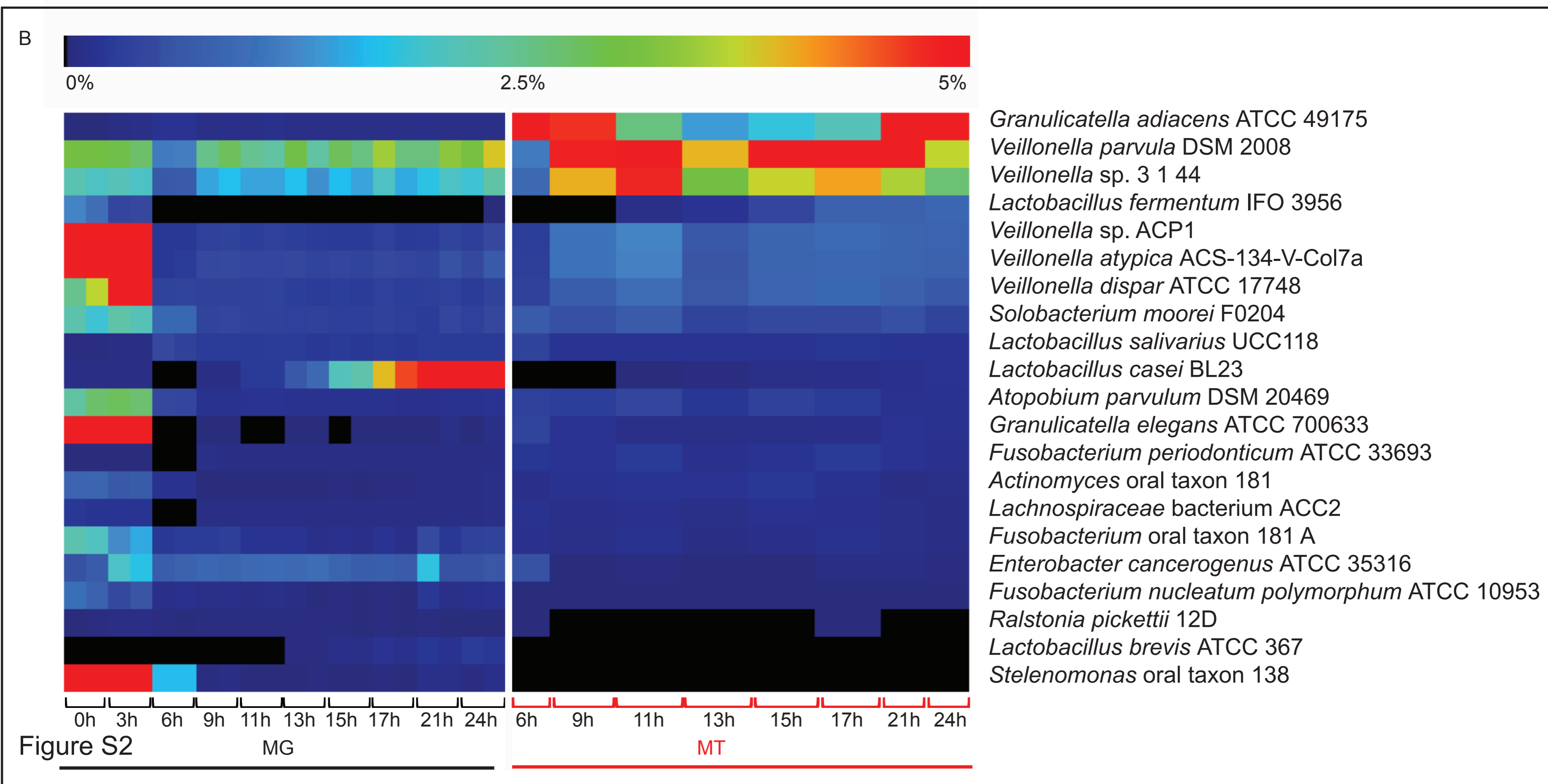

Figure S2

Supplement: Supplementary file 2 — Figure S1. Distribution of key bacterial taxa in the biofilm community obtained by DNA and mRNA deep sequencing approaches, respectively. Heat maps show relative abundance estimations of DNA, and mRNA reads representing the most abundant reference genomes. DNA read mapping was performed for all stages of growth (0 to 24 h, pH 7.2 to 4.3). mRNA read mapping was performed with libraries representative of 6–24 h of growth (pH 5.5 to 4.3). Two replicate DNA libraries were prepared from each growth stage, while three replicate mRNA libraries were prepared, except from six hours, 21 h, and 24 h of growth, for which two libraries were prepared. Metagenomics (MG) and metatranscriptomics (MT) read mapping results are shown for the most abundant Streptococcus species in panel A while other bacterial taxa are presented in panel B. (PDF 1603 kb) [file 40168_2018_591_MOESM2_ESM.pdf]
