# Supplementary material for: Uncovering complex microbiome activities via metatranscriptomics during 24 hours of oral biofilm assembly and maturation
Source: Microbiome. 2018 Dec 6;6:217. doi: 10.1186/s40168-018-0591-4 (PMC6284299; doi:10.1186/s40168-018-0591-4)
Supplement: Supplementary file 18 — Figure S2. A putative sactipeptide biosynthetic gene cluster that was identified in Veillonella parvula genomes. The gene cluster was identified by using the antiSMASH program available at https://antismash.secondarymetabolites.org/. It was actively transcribed between six hours and 24 h of growth when pH dropped below 5.5 in both genomes. Genes are color coded based on putative function. The six-cysteine peptide SCIFF and Radical SAM motifs represent core modules of this group of natural products. (PDF 102 kb) [file 40168_2018_591_MOESM18_ESM.pdf]

Figure S2

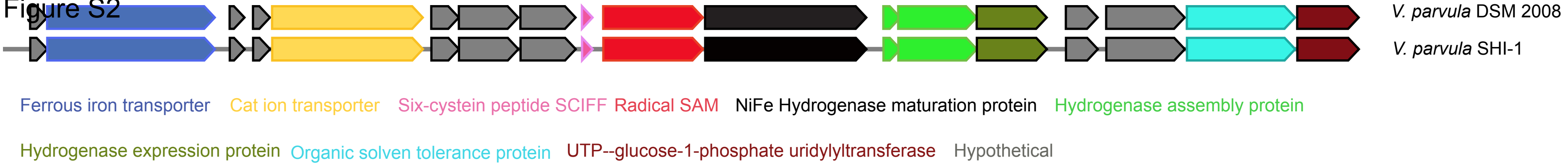
V. parvula DSM 2008
V. parvula SHI-1
Ferrous iron transporter
Cat ion transporter
Six-cystein peptide SCIFF
Radical SAM
NiFe Hydrogenase maturation protein
Hydrogenase assembly protein
Hydrogenase expression protein
Organic solven tolerance protein
UTP--glucose-1-phosphate uridylyltransferase
Hypothetical
